# Astroblastomas exhibit radial glia stem cell lineages and differential expression of imprinted and X-inactivation escape genes

**DOI:** 10.1038/s41467-022-29302-8

**Published:** 2022-04-19

**Authors:** Norman L. Lehman, Nathalie Spassky, Müge Sak, Amy Webb, Cory T. Zumbar, Aisulu Usubalieva, Khaled J. Alkhateeb, Joseph P. McElroy, Kirsteen H. Maclean, Paolo Fadda, Tom Liu, Vineela Gangalapudi, Jamie Carver, Zied Abdullaev, Cynthia Timmers, John R. Parker, Christopher R. Pierson, Bret C. Mobley, Murat Gokden, Eyas M. Hattab, Timothy Parrett, Ralph X. Cooke, Trang D. Lehman, Stefan Costinean, Anil Parwani, Brian J. Williams, Randy L. Jensen, Kenneth Aldape, Akshitkumar M. Mistry

**Affiliations:** 1 Department of Pathology and Laboratory Medicine, University of Louisville, Louisville, KY 40202 USA; 2 Department of Biochemistry and Molecular Genetics, University of Louisville, Louisville, KY 40202 USA; 3 The Brown Cancer Center, University of Louisville, Louisville, KY 40202 USA; 4 Institut de Biologie de l’ENS (IBENS), Inserm, CNRS, École Normale Supérieure, PSL Research University, Paris, France; 5 Department of Biomedical Informatics, The Ohio State University, Columbus, OH 43210 USA; 6 Department of Biomedical Informatics, University of Arkansas for Medical Sciences, Little Rock, AR 72205 USA; 7 Center for Biostatistics, Department of Biomedical Informatics, The Ohio State University, Columbus, OH 43210 USA; 8 NanoString Technologies, Seattle, WA 98109 USA; 9 Department of Cancer Biology, The Ohio State University, Columbus, OH 43210 USA; 10 Solid Tumor Translational Science, The Ohio State University, Columbus, OH 43210 USA; 11 Laboratory of Pathology, Center for Cancer Research, National Cancer Institute, Bethesda, MD 20892 USA; 12 Department of Pathology and Laboratory Medicine, Nationwide Children’s Hospital, Columbus, OH 43205 USA; 13 Department of Pathology, The Ohio State University, Columbus, OH 43210 USA; 14 Department of Pathology, Microbiology and Immunology, Vanderbilt University, Nashville, TN 37232 USA; 15 Department of Pathology and Laboratory Services, University of Arkansas for Medical Sciences, Little Rock, AR 72205 USA; 16 Department of Pathology and Anatomic Sciences, University of Missouri, Columbia, MO 65212 USA; 17 Department of Family and Community Medicine, Contra Costa County Health System, Martinez, CA 94553 USA; 18 Department of Pathology, Banner Gateway Medical Center, MD Anderson Cancer Center, Tempe, AZ 85284 USA; 19 Department of Neurosurgery, University of Louisville, Louisville, KY 40202 USA; 20 Department of Neurosurgery, University of Utah, Salt Lake City, UT 84132 USA; 21 Department of Neurological Surgery, Vanderbilt University, Nashville, TN 37232 USA

**Keywords:** CNS cancer, Neural progenitors, Epigenetics and plasticity, Cancer epigenetics

## Abstract

Astroblastomas (ABs) are rare brain tumors of unknown origin. We performed an integrative genetic and epigenetic analysis of AB-like tumors. Here, we show that tumors traceable to neural stem/progenitor cells (radial glia) that emerge during early to later brain development occur in children and young adults, respectively. Tumors with *MN1*-*BEND2 fusion* appear to present exclusively in females and exhibit overexpression of genes expressed prior to 25 post-conception weeks (pcw), including genes enriched in early ventricular zone radial glia and ependymal tumors. Other, histologically classic ABs overexpress or harbor mutations of mitogen-activated protein kinase pathway genes, outer and truncated radial glia genes, and genes expressed after 25 pcw, including neuronal and astrocyte markers. Findings support that AB-like tumors arise in the context of epigenetic and genetic changes in neural progenitors. Selective gene fusion, variable imprinting and/or chromosome X-inactivation escape resulting in biallelic overexpression may contribute to female predominance of AB molecular subtypes.

## Abstract (web summary)

Astroblastoma (AB) is an uncommon brain tumour and its origin remains unknown. Here, the authors perform integrative molecular analysis of 35 AB-like tumours and provide evidence that these arise in the context of epigenetic and genetic changes in neural progenitors occurring during brain development.

## Introduction

**Fig. 1:**
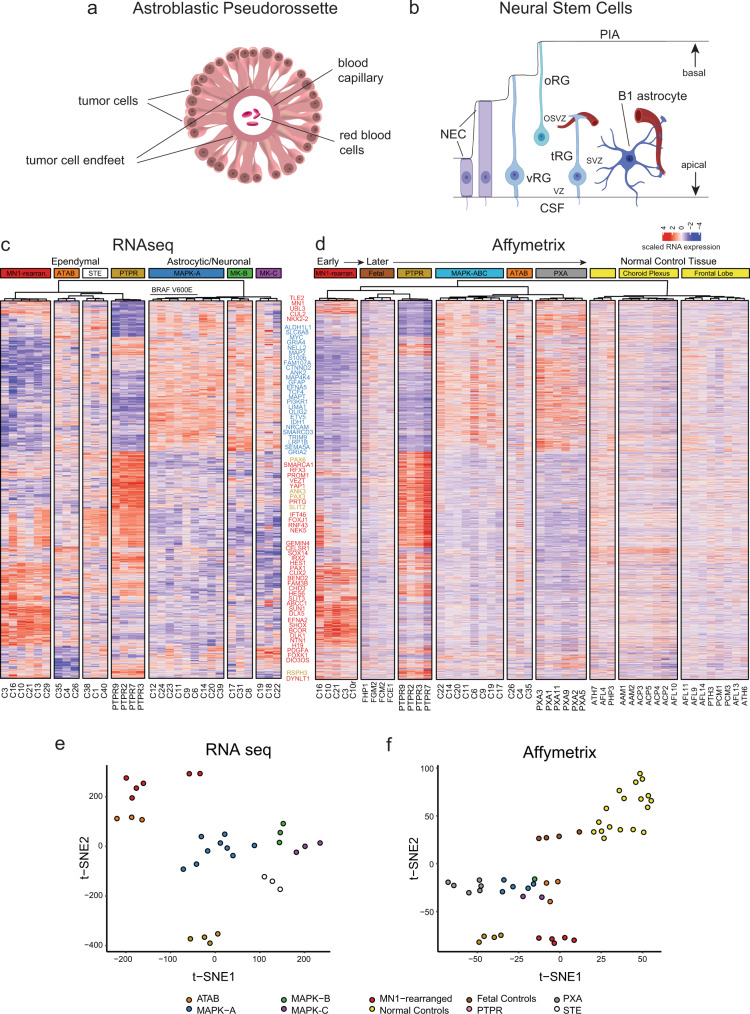
Astroblastoma and neural stem/progenitor cell morphologies and AB-like tumor molecular subtypes. **a** Classic astroblastic pseudorossette. **b** Neural stem cell morphologies. NEC, neuroepithelial cell; vRG, ventricular  zone radial glia cell; oRG, outer radial glia cell; tRG, truncated radial glia cell; VZ, ventricular zone; SVZ, subventricular zone; OSVZ, outer subventricular zone. **c** Unsupervised hierarchical clustering of RNAseq expression data separates AB-like tumors into *MN1-*rearranged and MAPK/PI3K pathway transcriptomic groups. Genes overexpressed in *MN1-BEND2* tumors are highlighted in red, those overexpressed in MAPK-ABC tumors in blue and PTPR in bronze. Some genes highly expressed in both *MN1-BEND2* tumors and PTPR are depicted in red. Abbreviations: AFL, adult frontal lobe; AAM, adult amygdala; ACP, adult choroid plexus; ATH, adult thalamus; FCE, fetal cerebrum; FCM, fetal cerebellum; FCG, fetal germinal matrix; FHP, fetal hippocampus; PCM, pediatric cerebellum, PHP, pediatric hippocampus; PTH, pediatric thalamus. **d** tSNE analysis of RNAseq expression data. **e** Hierarchical clustering and **f** tSNE analysis of Affymetrix HTA2 RNA expression data. Classical PXA tumors cluster separately from MAPK-ABC AB-like tumors demonstrating that they are separate biologic entities. Source data: Supplementary Fig. 1, Gene Expression Omnibus (GEO) accession numbers GSE165351, GSE165813.

Astroblastomas (ABs) are uncommon and historically controversial CNS neoplasms characterized by tumor cells surrounding a central capillary forming a flower petal-like structure called the *astroblastic pseudorosette* (Fig. 1a)^1–3^. AB-like tumors typically occur in the cerebral hemispheres of children and young adults and show considerable female predominance^3–5^. Their clinical behavior ranges from frequently surgically-amenable benign lesions to occasionally malignant. A long-standing question is whether ABs are of astrocytic or ependymal lineage^2^.

AB-like tumors are comprised of at least two genetic types based on the presence of the pV600E mutation of the B-Raf serine/threonine kinase gene (*BRAF*^*V600E*^)^2,4,6^ or rearrangement of the meningioma (disrupted in balanced translocation) 1 (*MN1*) gene^4,6^. Fusions between *MN1* at chromosome region 22q12.1 and BEN domain containing 2 (*BEND2*) at Xp22.13 are reported in *MN1*-rearranged tumors^6^.

Embryonic NSCs include highly proliferative neuroepithelial cells (NEC), which comprise the primitive neural tube and give rise to early neuronal progenitors (neuroblasts). Around five post-conception weeks (pcw) NEC begin to transform into ventricular zone radial glia (vRG), bipolar cells whose apical and basal processes contact the ventricular and pial surfaces of the developing brain, respectively^7,8^ (Fig. 1b). NEC and vRG exhibit interkinetic nuclear migration (IKNM), which helps maintain the tightly packed cellular structure of the ventricular lining during cell division. Like NEC, vRG can directly give rise to neuronal progenitors through asymmetric division. Further such vRG divisions produce B1 astrocytes (future adult subventricular zone NSCs), followed by ependymal cells^9^. The latter replace vRG in lining the brain ventricles.

vRG also produce outer radial glia (oRG), whose nuclei reside in the outer subventricular zone (OSVZ), and which appear at about 12 pcw^10^. Unlike vRG, oRG lack a prominent apical process and demonstrate mitotic somal translocation (MST) in which the cell body migrates basally prior to mitosis, facilitating expansion of the OSVZ and cortical plate^11^. They generate intermediate progenitor (IP) cells capable of further division termed transiently amplifying progenitors which generate the majority of cortical neurons. Lastly, oRG also give rise to astrocytes and oligodendrocytes.

Nowakowski et al.^12^ observed that the long basal processes of vRG become truncated in the human fetus at about 15 pcw. Unlike vRG and oRG, truncated radial glia (tRG) extend their basal processes only into the OSVZ where their end-feet typically surround blood capillaries similar to classic astroblastoma cells^1,2^ (Fig. 1a, b).

DNA methylation in the human brain is dynamic during development. Some genes on the inactive X chromosome in females can escape X inactivation^13–16^. These X-inactivation escape (XIE) genes, can acquire promoter hypomethylation in somatic cells resulting in biallelic expression from both X chromosomes, potentially doubling their gene dosage^13^.

Here we show that AB-like tumors with *MN1-BEND2* fusions appear to exclusively occur in females, and nearly always in children. They exhibit overexpression of *IGF2-H19* and *DLK1-DIO3* region, and other imprinted genes, XIE genes, and genes highly expressed during brain development prior to 25 pcw, including genes enriched in vRG and ependymal tumors. Other AB-like tumors overexpress mitogen-activated protein kinase (MAPK) pathway, phosphatidylinositol-3-kinase (PI3K) pathway and alternate XIE genes, and tend to occur in young adults of both sexes depending on molecular subtype. These tumors highly express oRG and tRG genes, and genes expressed after 25 pcw that are often involved in neuronal or astrocytic differentiation. Our findings strongly support that AB-like tumors arise in the context of epigenetic and genetic changes in neural progenitors during brain development: early ependymal tumors with *MN1-BEND2* from early vRG-derived cells, and MAPK pathway tumors demonstrating more classic astroblastoma histomorphology from oRG- or tRG-derived progenitors, respectively. Data also suggest that increased gene fusion, variable  imprinting, and XIE may contribute to the female predominance of certain AB-like tumor molecular subtypes. Lastly, we found that like *MN1-BEND2* tumors, nascent immature ependymal cells express IGF2 and may represent an important source of this growth factor in the fetal lateral ventricular zone niche.

## Results

### Tumors with AB histology segregate into distinct subtypes based on transcriptomic and mutational profiles

We performed RNAseq on 24 supratentorial AB-like cases with available material, including six *BRAF*^*V600E*^-mutant tumors^2^, six *MN1*-rearranaged tumors^4^ and twelve tumors without these genetic lesions (Table 1). Three tumors diagnosed as supratentorial ependymoma (STE) and four papillary tumor of the pineal region (PTPR)^17^ were also studied for comparison. Affymetrix gene microarray (HTA-2) expression analysis was additionally used to study 15 of the above AB-like tumors, one *MN1*-rearranaged tumor recurrence (C10r), the four PTPR, six histologically classic pleomorphic xanthoastrocytomas (PXA), four fetal brain samples (28–30 pcw) and 14 normal pediatric and adult brain samples.

**Table 1:** Patient demographic and tumor pathology data.

			Case No.	Age	Gender	Location	Ki-67%	Mitoses (per 10 hpf)	Necrosis	Survival (months)
***MN1*-rearranged**	MN1-BEND2		C3	16	F	L Parieto-occipital	20	3	+	138^a^
			C10	12	F	R Frontoparietal	4	0	−	147^a^
			C13	9	F	R Frontoparietal	25	14	+	68^a^
			C21	4	F	L Parieto-occipital	15	3	+	131^a^
			C29	18	F	L Parietal	5	1	−	227^a^
			C44	9	F	R Parietal	NA	NA	+	48^a^
			C7^b^	33	F	L Temporal	10	3	+	120^a^
			C43^b^	10	F	R Parietal	10	6	+	120^a^
			C16	8	M	L Temporal	0	0	−	144^a^
**MAPK Supercluster**	**MAPK-A**	BRAF-V600E	C6	33	F	R Frontal	5	2	−	59
		BRAF-V600E	C9	38	F	R Frontal	10	2	−	123
		BRAF-V600E	C11	25	F	R Occipital	20	4	+	141^a^
		BRAF-V600E	C12	12	F	R Frontal	17	3	+	2
		BRAF-V600E	C23	20	F	R Temporal	22	3	+	15^a^
		BRAF-V600E	C24	20	F	L Parietal	10	2	−	79^a^
			C14	71	F	L Occipital	4	5	+	18
			C20	55	M	L Parietal	25	2	+	40
			C39	20	F	L Parietal	35	11	+	132^a^
	**MAPK-B**		C8	40	F	R Temporal	NA	7	−	168^a^
			C17	20	F	L Occipital	10	3	+	279^a^
			C31	28	F	R Frontal	6	0	−	25^a^
	**Group C**		C18	9	M	L Parasagittal	3	0	−	222^a^
			C19	36	M	R Frontal	45	10	+	252^a^
			C22	9	M	R Parietal	15	2	−	47
			C27	42	M	NA	NA	0	−	NA
**Others**	**STE**		C1	10	F	L Parietal	1	0	−	208^a^
			C30^b^	19	M	L Frontal	60	10	+	12^a^
			C38	14	M	R Temporal	20	3	+	154^a^
			C40	25	F	R Frontal	4	0	−	67^a^
	**ATAB**		C4	44	F	L Temporo-occipital	25	75	−	NA^c^
			C26	58	F	R Parietal	NA	5	+	184
			C35	76	M	L Frontotemporal	10	5	+	7
	**NTD**		C15	40	M	R Temporal	10	5	−	2
			C41	57	M	Temporal	14	3	+	28
			C42	19	M	L Temporal	2	0	−	14^a^

Supratentorial ependymoma (STE) were RELA fusion tumors except C38. Case 30 has no transcriptional data, however is documented to contain the ZFTA-RELA fusion by FISH. Case 27 has no transcriptional data, but like the MAPK-B tumor C8, clustered with Group C tumors by DNA methylation. C16 clustered with MN1-BEND2 tumors by RNA analysis but not DNA methylation.

Cases are grouped based on transcriptomic clustering in Fig. 1c, unless otherwise specified.

*NA* not available, *NTD* no transcription data.

^a^The patient was alive at the most recent follow-up.

^b^No transcription data but grouped by DNA methylation analysis.

^c^The patient is deceased however the length of survival is not available.

Dimensionality reduction of the two expression datasets was then performed individually using tSNE (23,937 mappable genes in the Affymetrix data and 24,917 mappable and encodable RNAseq gene transcripts). Next, we identified 1793 genes differentially expressed among the resultant expression clusters that were common to both datasets using analysis of variance (*P* < 0.05).

**Fig. 2:**
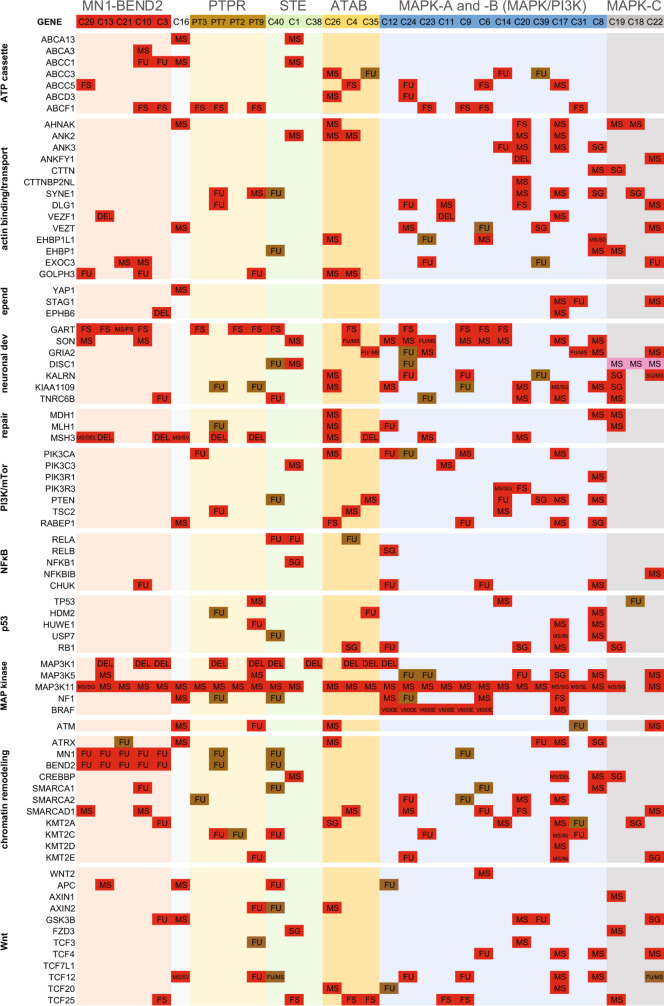
Gene mutations and fusions segregate AB-like tumor genetic groups. Pathway gene alterations across AB-like tumors, ATAB, PTPR and RELA STE are grouped. Missense = MS, splice region variant = SV, stop gained = SG, stop lost = SL, frameshift = FS, deletion = DEL, insertion = INS, fusion = FU, PT = PTPR. Detected genetic alterations are highlighted in red. Brown cells indicate fusions detected by only one read. Pink indicates a FathmmMLK score <0.5, but predicted to be damaging by PolyPhen or deleterious by SIFT. Source data: Supplementary Data 1 and 2.

Unsupervised hierarchical clustering of these 1793 genes using the RNAseq data yielded seven tumor clusters (Fig. 1c), similar to tSNE (Fig. 1e). Tumors with *MN1* rearrangement, including five containing the *MN1-BEND2* gene fusion (Table 1), formed a discrete RNA cluster, as did all *BRAF*^*V600E*^-mutant tumors combined with three tumors without *BRAF*^*V600E*^. The latter showed mutations of other MAPK, and frequently PI3K/AKT/mTOR pathway genes (Fig. 2, Supplementary Data 1). We designated this RNA cluster MAPK-A. Three additional tumors without *BRAF*^*V600E*^ mutation, but otherwise showing similar mutation profiles as MAPK-A tumors, closely clustered with them and were designated MAPK-B (Fig. 1c, e). Hence forth, we refer to these two groups collectively as MAPK-AB lesions. Three tumors lacking both *BRAF*^*V600E*^ mutation or obvious PI3K pathway mutations, designated MAPK-C, also formed a subgroup within the MAPK supercluster (MAPK-ABC).

Three histologically atypical AB-like tumors (ATAB) also formed a transcriptomic cluster (Fig. 1c, e). These occurred in older adults and showed high-grade histology (Table 1). Lastly, three STEs (two containing the *ZFTA-RELA* fusion), and the four PTPR grouped into respective RNA clusters.

### MN1-BEND2 containing tumors, MAPK supercluster astroblastomas, and pleomorphic xanthoastrocytomas (PXAs) are genetically distinct entities

Hierarchical clustering of the Affymetrix HTA-2 data using the same 1,793 genes resulted in nine clusters: 1) *MN1*-rearranged tumors; 2) fetal brain control tissue; 3) PTPR; 4) MAPK-ABC tumors; 5) ATAB; 6) PXA; and 7-9) normal adult and pediatric brain control tissue (Fig. 1d, f). Although *BRAF*^*V600E*^-mutant AB-like tumors loosely group with PXA by genomic DNA methylation^4^, all AB-like tumors clustered separately from PXA in hierarchical clustering and tSNE analyses of tumor transcriptomes. Notably, gene expression in normal control tissues was markedly different from that of tumors (Fig. 1d). These data indicate that, albeit likely ontologically related^2^, MAPK-ABC tumors are genetically distinct from PXA.

### Neural stem cell and early ependymal genes are highly expressed in MN1-BEND2 tumors

*CUX2, SHOX, SOX14, IRX2, PAX1, HOXD10, DLX5, and PRRX2* are homeobox genes relatively overexpressed in *MN1-BEND2* tumors (Fig. 1c, d and Supplementary Figs. 1–3). Interestingly, the fetal growth factor gene *insulin growth factor 2* (*IGF2*) and the ependymal fate determining gene *FOXJ1*^9^, both markers of intracranial ependymoma^18^, were relatively overexpressed by *MN1-BEND2* tumors, as well as STE (Supplementary Figs. 1, 2). *MN1-BEND2* tumors did not overexpress the STE-associated and neuronal marker gene *ANK2*^19,20^. However, *MN1-BEND2* tumors did overexpress *CELSR1*, encoding a planar cell polarity protein necessary for neural tube closure^21^ and maintenance of intercellular ciliary basal body (centriole) alignment in vRG and ependymal cells^22^. They also highly expressed *SUN1*, encoding a protein required for IKNM in NEC and vRG^23^ and *CHD3*, a part of the *Mi-2/NuRD histone deacetylase complex* (Fig. 1c, d).

### MN1-BEND2 tumors and PTPR share ependymal gene expression and low expression of oRG, glial and neuronal genes

*SOX14*, *IRX2*, *CELSR1, KCNJ5* encoding the inward rectifier potassium channel Kir3.4, and *TFF3* encoding an epithelial cell mobility protein, were overexpressed by *MN1*-rearranged tumors and PTPR almost exclusively (Fig. 1c, d and Supplementary Figs. 1–3). Both also strongly expressed *FOXJ1*^24,25^ and the additional ependymoma-associated genes *RFX3*, encoding a transcription factor essential for development of motile cilia^26^, *YAP1*^27^ and *NELL2* (Supplementary Figs. 1–3). Overall, these findings argue for ependymal differentiation of both *MN1-BEND2* tumors and PTPR. The latter differ from *MN1-BEND2* tumors and many STEs, however, in that they are *IGF2* relative under-expressers. This may be related to their 3rd ventricular diencephalic derivation, in contrast to the likely telencephalic origin of *MN1-BEND2* tumors and STE. Moreover, the fact that PTPR more highly express the NSC markers PAX6 and OTX2 argues they may be more closely related to a RG stem-like cell^28^, perhaps derived directly from such a cell and lacking an IP possibly required in the genesis of *MN1-BEND2* tumors and STE.

In contrast to MAPK supercluster tumors, *MN1*-rearranged tumors, PTPR and STE showed relatively low expression of the neuronal genes *GRIA2 (glutamate ionotropic receptor AMPA type subunit 2*), *MAOA* (*monoamine oxidase A*, an XIE gene) and *MAPT (microtubule associated protein tau)*. They also demonstrated low expression of the astrocyte markers *OLIG2*, *GFAP, S100β* and *ALDH1*, the oRG markers *IDH1* and *TNC*^10^, and other genes highly expressed in MAPK-ABC tumors, *e.g*., *TCF4, LRP1B*, *CTNND2, NCAN*, *DISC1*, *HIF1A*, *MYCBP2*, *ABCC5,* and *ANK2*. PTPR and STE however do overexpress some genes also highly expressed in MAPK supercluster tumors, but not *MN1*-rearranged tumors (Fig. 1c, d, Supplementary Figs. 1–3).

TCF4 is a WNT transcription factor. *LRP1B* and *CTNND2* are WNT pathway regulatory genes^29^. The latter encodes δ-catenin 2, which affects dendritic spine and synapse formation, and is a marker of oRG and neurons^10^. *DISC1* encodes disrupted in schizophrenia 1, which is involved in neurogenesis and astrogenesis, and is a positive regulator of WNT signaling and negative regulator of PI3K/AKT/mTOR signaling^30,31^. The relatively high expression of these genes in MAPK-ABC tumors suggests that the WNT pathway is important in their biology.

### MN1-BEND2 and MAPK supercluster tumors show characteristic mutational profiles

In addition to indel, stop gain and stop lost mutations, only missense or splice site mutations found to be “deleterious” by SIFT, “possibly or probably damaging” by PolyPhen and/or deleterious by FathmmMKL^32^ (defined as FathmmMKL scores above 0.5), are considered herein unless otherwise specified (Supplementary Data 1). *MN1*-rearranged tumors demonstrated multiple recurrently mutated genes, including *ABCC1*, *IGF2*, *ABCF1*, and *MSH3* (Fig. 2). *ABCC1* encodes the ATPase cassette transporter protein multidrug resistance 1, a stem cell marker and MYCN transcriptional target conferring resistance to chemotherapeutics. An *ABCC1-SSU72* fusion was present in case C10, and *ABCC1-LUCAL3* and *ABCC1-ZHHC14* fusions were found in C3 (Fig. 2, Supplementary Data 2). A heterozygous *ABCC1* single nucleotide substitution (SNS), p1088P/L was present in the *MN1*-rearanged tumor lacking *MN1-BEND2* fusion from a male patient (C16). Predicted deleterious *ABCC1* mutations were additionally noted in *ZFTA-RELA* STE and one ATAB in which *ZFTA-RELA* was detected by a single read (C4). *ABCC1* was also relatively overexpressed in *MN1*-rearanged and *ZFTA-RELA* tumors (Fig. 1c, d and Supplementary Figs. 1, 2), suggesting they share a common ependymal stem cell lineage. The mismatch repair gene *MSH3* was mutant in four of six *MN1*-rearranged cases.

Mutations in PI3K/AKT/mTOR pathway genes, *e.g*., *PIK3 subunits*, *PTEN*, *TSC2* and *NF1*, were found in most MAPK-AB and ATAB tumors (Fig. 2). Deleterious mutations of the *NF1* tumor suppressor gene occurred in three MAPK-AB tumors, the *MN1-BEND2-negative MN1*-rearranged tumor, and an ATAB. PI3K/AKT/mTOR pathway gene mutations were notably absent in *MN1-BEND2* and MAPK-C tumors.

*TCF4* was mutant in two MAPK-B tumors and one MAPK-C tumor, and involved in a fusion in a *BRAF*^*V600E*^-mutant MAPK-A tumor (Fig. 2, Supplementary Data 1, 2). *ANK2* and *ANK3* mutations were found in non-*BRAF*-mutant MAPK-AB tumors. The *BRAF*^*V600E*^-mutation negative case C14 occurred in the oldest patient in the MAPK-A group. This case showed multiple PI3K pathway alterations, including a *TSC2* mutation, *PTEN-ANK3* fusion and an out-of-frame *RP5-864K19.4-TRIM27* fusion. *TRIM27* encodes an E3 ligase that ubiquitinates phosphatidylinositol-4-phosphate 3-kinase catalytic subunit type 2 beta (PIK3C2B)^33^. Its disruption may thus lead to increased PI3K signaling.

### MAPK-C and other non-MN1-BEND2, non-BRAF^V600E^–harboring lesions demonstrate unique mutations

MAPK-C tumors occurred in males and showed non-fusion mutations in genes involved in actin and adherens junction binding, including *KALRN*, *TRIO*, *DISC1*, and *ANK3* (Fig. 2, Supplementary Data 1–3). *KALRN* and *TRIO* encode Rho guanine nucleotide exchange factors, which are downstream effectors of noncanonical WNT signaling^34,35^. *KALRN, TRIO*, and *DISC1* are involved in synapse formation and plasticity, axonal growth and adolescence-associated dendritic spine pruning^36,37^. Interestingly, fusions involving *KALRN or TRIO* were only detected in *BRAF*^*V600E*^-mutant MAPK-A and STE tumors *from females*, e.g., *KALRN-SESN3*, *KALRN-PLXND1 and TRIO-NDUFV3*, and *TRIO-DNAH5* and *TTC21B-KALRN*, respectively. *DISC1* was mutant in MAPK-C and STE tumors.

*PARP8*, *PTEN, ANK2*, *ANKFY1,* and *KIAA1109* non-fusion mutations were unique to non-*MN1-BEND2*, non-*BRAF*^*V600E*^ tumors. *KIAA1109* is important in cortical development^38^ and mutant in glioblastoma^39^. *ANK* genes encode *ankyrins*, which link membrane proteins to the actin cytoskeleton and are involved in dendritic spine growth and synaptic plasticity^40^.

Mutations in *CTTN* (*cortactin*) or *CTTNBPNL* (*cortactin-binding protein 2 N-terminal–like*) occurred in *non-BRAF*^*V600E*^ MAPK-ABC tumors. Cortactin is an actin binding protein involved in cytoskeletal remodeling and implicated in tumor invasion and WNT-dependent synaptic plasticity^41,42^. Three tumors contained *VEZT* mutations. Its protein product vezatin binds the cadherin-catenin complex linking adherens junctions to the actin cytoskeleton^43^.

The most frequently mutated gene in non-*MN1-BEND2*, *non-BRAF*^*V600E*^ tumors was *AHNAK*, mutated in six such tumors, including MAPK-C tumors. *AHNAK* encodes neuroblast differentiation-associated protein, a cadherin-associated actin binding protein implicated in neural cell differentiation, migration, and tumor metastasis^44^.

### DNA methylome analysis reveals a distinct methylation class for MAPK-C tumors

**Fig. 3:**
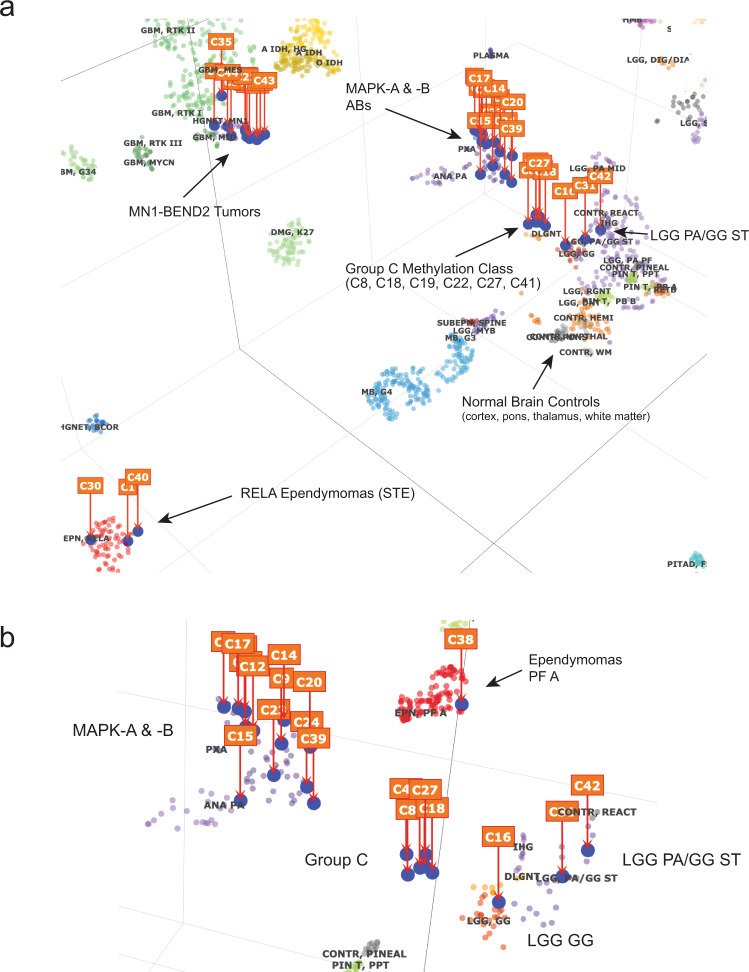
Unsupervised tSNE analysis of genomic DNA methylation data reveals a distinct tumor DNA methylation class for Group C tumors. **a** 3-dimensional tSNE plot of all study samples with the German Cancer Center (DKFZ) reference sample set. **b** Enlargement of MAPK/PI3K (MAPK-A & -B) and Group C methylation classes. 32,000 methylation sites were used for the analyses. LGG ST PA/GG, low-grade glioma, supratentorial pilocytic astrocytoma/ganglioglioma (nonspecific methylation class); LGG GG, low-grade glioma, ganglioglioma methylation class. Source data: GEO GSE125450 and GSE166569.

We performed genomic DNA methylation analysis of twelve AB-like tumors not previously analyzed. When these data were combined with that from our prior study^4^, tSNE analysis revealed that all *MN1*-*BEND2* tumors clustered with the methylation class “MN1 high-grade neuroepithelial tumor (MN1-HGNET)” (Fig. 3).

MAPK-A AB tumors containing the *BRAF*^*V600E*^ mutation again loosely grouped with the “pleomorphic xanthoastrocytoma (PXA)” methylation class^4^ (Fig. 3). BRAF-wildtype MAPK-A tumors C14, C20 and C39, and an additional AB-like tumor not studied by RNAseq (C15), appeared on the periphery of the PXA methylation cluster. The three MAPK-C transcriptional cluster tumors, one MAPK-B tumor (C8) and two tumors not studied by RNAseq were among six cases (C8, C18, C19, C22, C27, and C41) forming a distinct genomic DNA methylation class, designated the Group C methylation class.

Of the remaining non-*BRAF*^*V600E*^ cases, including the *MN1*-rearranged tumor lacking *MN1-BEND2* (C16), three grouped with the nonspecific “low-grade glioma/supratentorial pilocytic astrocytoma/ganglioglioma” methylation class^6^, and C1 containing the *ZFTA-RELA* fusion, grouped with RELA ependymomas. A STE lacking *ZFTA-RELA* grouped with posterior fossa type A ependymomas by DNA methylation, and two ATAB grouped with glioblastoma subtypes (Fig. 3).

### AB-like tumor histology is characterized by astroblastic pseudorosettes, and oRG/tRG-like classic astroblastoma cytomorphology in Group C tumors

**Fig. 4:**
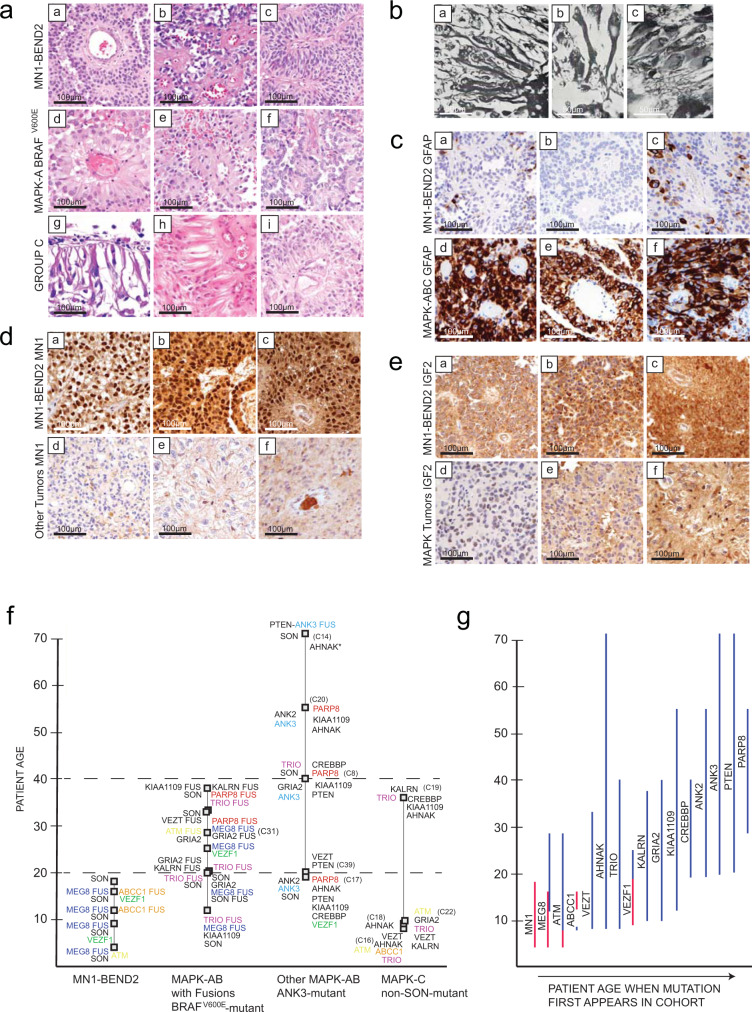
Astroblastoma-like tumor subtypes exhibit characteristic histologies and genetic alterations. **a** AB tumor histology is characterized by *astroblastic pseudorosettes*, and oRG-like morphology in Group C tumors. **a**–**i** H&E-stained AB-like tumor cases. Top row *MN1-BEND2* cases; Middle row *BRAF*^*V600E*^*-*mutant MAPK-A cases*;* Bottom row Group C methylation class tumor cases. Subpial accumulations of tumor cells in C22 (**g**) demonstrated long basal processes extending to the pial surface and inconspicuous apical processes, morphologically resembling outer radial glial^10^. Similar cells extend basal processes to blood vessels in C27 and C19 (**h**–**i**). **b** Astroblastoma tumors cells within astroblastic pseudorosettes from Bailey and Bucy’s^1^ original cohort. **a**, **b** Tumor cells with long basal processes resembling those in Group C tumors (Plate III, Figs. 1. and 2). **c** Tumor cells from a case more similar in appearance to those in MAPK-AB tumors (Plate IV, Fig. 1). Mallory-Davidoff stain (**a**, **b** 850× and **c**, 600×)^1^. Reproduced with the publisher’s permission. **c** *MN1-BEND2* and MAPK-ABC AB-like tumors show characteristic GFAP immunoreactivity. **a**–**c** Only focal staining for GFAP is observed in *MN1-BEND2* tumors. **d**–**f** MAPK-ABC tumors are strongly and diffusely GFAP immunoreactive. **d** MN1 diffusely highlights tumor nuclei in all *MN1-BEND2* tumors tested (**a**–**c**). Significant nuclear MN1 immunoreactivity is absent in non-*MN1-BEND2* AB cases (**d**–**f**). **e** *MN1-BEND2* tumors showed strong granular cytoplasmic IGF2 immunostaining (**a**–**c**), not seen in non-*MN1-BEND2* AB-like tumors (**d**–**f**). Immunohistochemical staining for all antibodies and samples was performed at least twice with equivalent results. **f**, **g** Mutations in AB-like tumor related genes occur in a patient age dependent manner. *ABCC1* mutations and fusions all occurred in patients 16 years old or less. Its highest expression in the Allen Human Developmental Transcriptome (AHDT) was before 25 pcw. *VEZT* and *VEZF* mutations and *CTD-2152M20.2-GOLPH3* fusions were all found in tumors from patients aged 25 and younger (Supplementary Data 2). The highest *GOLPH3*, *VEZT* and *VEZF1* expression in the AHDT was under 25 pcw. *MEG8* fusions occurred only in females 30 years of age and younger, most below 16 years, and only in *MN1-BEND2* and MAPK-A tumors. *GRIA2* mutations were present in MAPK-ABC cases. *ANK3*, *PARP8* and *PTEN* alterations were mostly present in non-*BRAF*^*V600E*^ MAPK-AB patients. A *PARP8* fusion was present in a *BRAF*^*V600E*^ tumor. Conversely, high *GRIA2* expression spans the fetal and postnatal AHDT, but not below approximately 12 pcw. *GRIA2* mutations are absent in *MN1*-rearranged tumors. Some genetic lesions were relatively specific to AB-like tumor genetic types: *MN1-BEND2* fusions and *ABCC1* mutation or fusion in *MN1*-rearranged tumors, and *TRIO* and *KALRN* fusions in *BRAF*^*V600E*^ tumors. SON mutations span the gamut of patient ages, as does its expression in the AHDT. These presumed somatic mutations were found in tumors clinically presenting at the indicated ages, however this data only indicates that the mutation was acquired in a patient sometime prior to that age, and some could have indeed been germline. Colors are arbitrary in **f**. In **g** *MN1-BEND2* tumor mutated genes are in red and MAPK-ABC tumor mutated genes are in blue. Source data: Supplementary Data 1 and 2.

It is not possible to precisely differentiate AB-like tumor genetic subtypes solely by histologic features, as tumor cytology is variable from cuboidal to tapered-columnar to spindle-shaped, often within the same tumor^2,4^. In general, however, *MN1-BEND2* tumor cellular morphology tends to be more cuboidal to stout-columnar and these tumors more often demonstrate vascular sclerosis, and lack eosinophilic granular material, in comparison to MAPK supercluster tumors^4^ (Fig. 4a [a–c]). MAPK-ABC tumors (Fig. 4a [d–i]) more frequently demonstrate tapering basal processes and inconspicuous or absent apical processes, especially Group C tumors (Fig. 4a [g–i]). The latter contained elongate monopolar cells whose endfeet contacted the brain pial surface or basal lamina of blood vessels mimicking oRG and tRG, respectively, similar to astroblastoma cells originally depicted by Bailey and Bucy^1^ (Fig. 4b [a, b]). Like MAPK-AB tumors, Group C tumors also demonstrated eosinophilic granular bodies and occasional multinucleated cells^2^, as previously noted in astroblastomas by Bailey and Bucy^1^.

### AB tumor subtypes show characteristic protein expression

*MN1-BEND2* tumors exhibited relative underexpression of GFAP mRNA (Fig. 1c, d, Supplementary Figs. 1–3). They were also predominantly GFAP negative by immunohistochemical staining or showed only patchy GFAP immunoreactivity (Fig. 4c [a–c]), similar to ependymomas. Conversely, MAPK-ABC tumors were diffusely GFAP immunoreactive (Fig. 4c [d–f]). Human vRG are present by 5 pcw and first become GFAP-positive by immunohistochemistry at about 11 pcw^45^. Thus, similar to *MN1-BEND2* tumors, *early* vRG do not show diffuse GFAP immunoreactivity.

*Strikingly*, all *MN1-BEND2* tumors demonstrated diffuse *nuclear* MN1 positivity (Fig. 4d [a–c]). Some MAPK-ABC tumors showed probable nonspecific *cytoplasmic* MN1 immunoreactivity; however, none exhibited diffuse MN1 nuclear immunoreactivity (Fig. 4d [d–f]). MN1 and BEND2 immunoreactivity were also confirmed by western blot in a *MN1-BEND2* case with available frozen tumor (C29) (Supplementary Fig. 4). *MN1-BEND2* tumors also demonstrated strong cytoplasmic IGF2 immunoreactivity (Fig. 4e [a–c]), which was absent or weak in non-*MN1-BEND2* AB-like tumors (Fig. 4e [d–f]).

### MN1-BEND2 and MAPK-ABC tumor gene expression overlaps that of NEC and vRG, and tRG and oRG, respectively

**Fig. 5:**
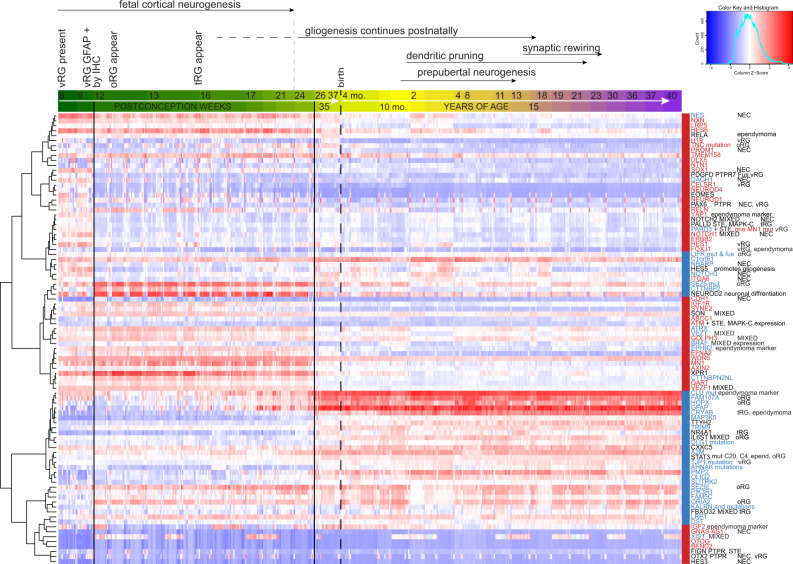
*MN1-BEND2* and MAPK-ABC tumor-associated genes demonstrate fetal age-specific expression and cluster with vRG and oRG marker genes, respectively. The developmental expression time course of NSC markers and select genes overexpressed or mutated in *MN1-BEND2* and MAPK-ABC AB-like tumors was obtained from the Allen Human Developmental Transcriptome database and subjected to unsupervised hierarchical clustering. *MN1-BEND2* tumor-associated genes (relatively overexpressed or mutated) are depicted in red and MAPK-ABC tumor associated genes are in blue. Genes associated with NEC, vRG, tRG and oRG are indicated. The x-axis age bar progressive color scheme is arbitrary. Transcript expression is normalized by reads per kilobase of transcript per million mapped reads (RPKM) to compensate for RNAseq generation of more sequencing reads from longer RNA molecules. Data is from up to 16 brain regions from 42 specimens (Allen Human Brain Atlas Developmental Transcriptome, Institute for Brain Science, available from: human.brain-map.org). The figure source data is available in the Source Data File.

We next compared expression of relatively overexpressed or mutated genes in *MN1-BEND2* versus MAPK-ABC AB-like tumors to that in the developing human brain utilizing the *Allen Brain Atlas Human Developmental Transcriptome* database^46^. Several transcripts selectively expressed or mutated in *MN1-BEND2* tumors, *e.g., MN1*, *IGF2*, *ABCC1,* and *VEZF1*, are more highly expressed in the fetal brain prior to 25 pcw (Figs. 4f, g, 5). Many such transcripts, e.g., *NXN*, *CTTNBP2NL* and *H19*, are highly expressed prior to 9 pcw when cellular proliferation in the developing brain peaks. Indeed, *H19* message is enriched in human vRG as determined by single cell RNAseq^47^. The period prior to 25 pcw corresponded to the highest initial expression of NEC genes within the database, e.g., *SOX1*, *DACH1*, *PROM1*, *NOTCH1*, *NOTCH2*, *ITGA6* and *NRARP*; vRG genes, e.g., *H19*, *FOXJ1* and *HES1*; the tRG gene *PALLD*, and a few oRG genes, e.g., *LIFR* and *SEZ6L*. In contrast, genes more highly expressed or mutated in MAPK-ABC tumors, *e.g., GFAP*, *BRINP3* and *TRIM9*, and certain oRG genes, e.g., *FAM107A* and *HOPX*, are more highly expressed subsequent to 25 pcw when genes related to dendritic spine and synapse development, *e.g*., *CLU*, *DLG1*, *KALRN*, *SLITRK2*, become more highly expressed^48^ (Fig. 5). The latter period corresponds to the end of widespread cortical neurogenesis and the beginning of cerebral gliogenesis. In contrast to genes highly expressed in *MN1-BEND2* tumors that tended to cluster with NEC and vRG marker genes, those selectively expressed in MAPK-ABC tumors tended to cluster with markers of vRG and oRG (Fig. 5). These data suggest that *MN1-BEND2* tumors are derived from cells closely related to early vRG that maintain high expression of many NEC genes, and that MAPK-ABC tumors are derived from cells related to oRG that retain expression of some vRG markers.

In order to further explore possible neural stem/progenitor cells which AB-like tumors might share ontologic lineage, we compared published neural stem/progenitor cell gene expression sets^10,49,50^ (Supplementary Data 4) to tumor gene expression using hierarchical clustering (Supplementary Fig. 5) and GSEA (Supplementary Fig. 6) NEC, general RG (gRG), vRG, tRG, and oRG signature genes were significantly upregulated in nearly all tumor types compared to normal pediatric and adult control brain tissues (based on our stringent family-wise error rate *P* value, p[FWER]) of ≤0.025). The only exceptions were vRG genes in PTPR and NEC genes in PXA (Supplementary Fig. 6A–D, Supplementary Data 5).

NEC genes were significantly enriched in *MN1-BEND2* tumors versus control fetal brain, and relatively enriched in *MN1-BEND2* tumors compared to PTPR, MAPK-ABC and PXA. The latter approached statistical significance (pFEWR = 0.028) (Supplementary Fig. 6E–H, Supplementary Data 5). *MN1-BEND2* tumors were relatively enriched in vRG genes compared to all other tumors, however, this was not statistically significant (Supplementary Fig. 6J–L).

The most significant and consistent GSEA findings for NSC genes were enrichment of PTPRs for tRG genes and PXAs for oRG genes. PTPR were significantly enriched for tRG gene expression compared to controls and all other tumor types, except PXA: to fetal brain (pFEWR < 0.0001), *MN1-BEND2* tumors (pFEWR = 0.004), ATAB (pFEWR = 0.001), MAPK-ABC (pFEWR = 0.002) and control pediatric and adult brain (pFEWR < 0.001) (Supplementary Fig. 6 M–P, Supplementary Data 5).

Both MAPK–ABC tumors and PXA were significantly enriched for only tRG and oRG compared to fetal brain (Supplementary Fig. 6Q, Supplementary Data 5). Compared to *MN1-BEND2* tumors, MAPK-ABC tumors were significantly enriched only for oRG genes, but showed more frequent mutation of tRG and oRG genes (Supplementary Fig. 7). PXA was enriched for tRG, and more so for oRG compared to *MN1-BEND2* and MAPK-ABC tumors (Supplementary Fig. 6P and T, pFEWER < 0.001, Supplementary Data 5). ATAB showed no clear stem/progenitor cell gene enrichment pattern likely attributable to the heterogeneity of this group.

### The MN1-BEND2 tumor markers MN1 and IGF2 are expressed by differentiating ependymal cells in the P0–P6 mouse ventricular zone

**Fig. 6:**
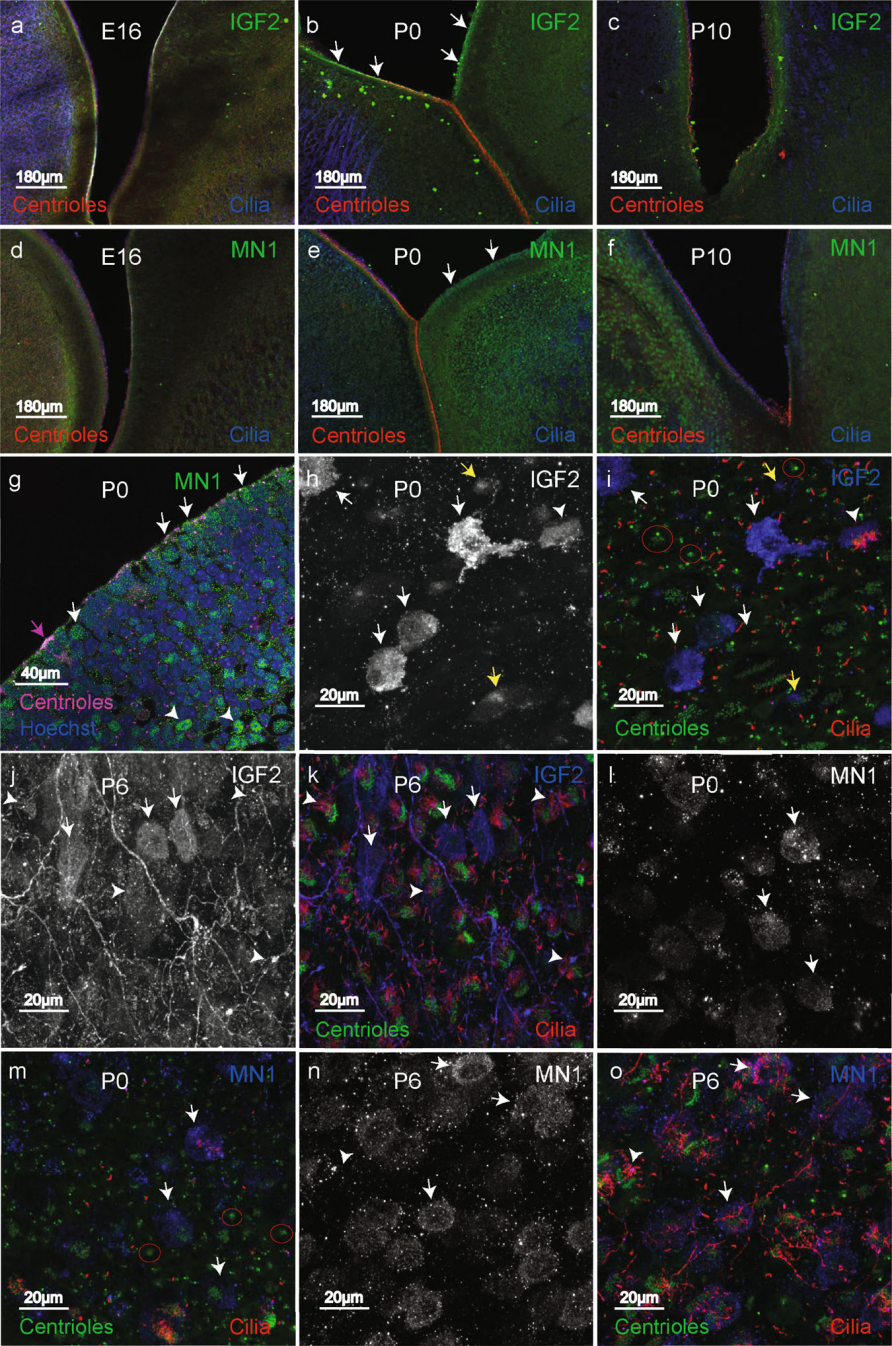
MN1 and IGF2 are expressed by differentiating ependymal cells in the P0 to P6 mouse ventricular zone. **a**–**g** Immunofluorescence stained embryonic (E16), newborn (P0) and postnatal day 10 (P10) mouse brain coronal sections are shown. **a**, **c**, **d** and **f** In E16 and P10 coronal sections MN1 and IGF2 staining is not apparent. **b** IGF2 staining is seen in the ventricular zone of the medial and lateral ventricular walls in P0 animal brains (arrows). **e** MN1 labels immature ependymal cells in the P0 lateral ventricular wall covering the striatum on the right (arrows). The medial wall ependyma are mature and demonstrate multiple centrioles (red) and motile cilia (blue). MN1 staining is not evident in these cells. **g** High power image of P0 lateral ventricular wall showing green MN1 staining of immature ependymal cell nuclei and cytoplasm (white arrows) and more basally located striatal cells (arrowheads), which could explain the cortical or juxtacortical location of *MN1-BEND2* tumors. The pink arrow indicates a more mature differentiating ependymal cell demonstrating multiple centrioles (pink fluorescence). **h**, **i** P0 whole mounts of the lateral ventricular wall show IGF2-positive microglia situated on the ependymal surface (arrows). An IGF2-positive, late differentiating ependymal cell with short multiple cilia is indicated by the arrowhead. Earlier stage differentiating ependymal cells with focal IGF2 staining are indicated by yellow arrows. Red circles highlight very early “halo stage” differentiating ependymal cells (**i**, **l**), which are IGF2-negative (**i**). **j**, **k** In P6 whole mount lateral wall, IGF2 again stains microglia (arrows) and immature ependyma with short cilia (arrowheads). **l**, **m** Mostly unciliated differentiating ependymal cells show MN1 staining in the P0 lateral wall (arrows). Very early halo stage nascent ependyma are MN1-negative (**l**). **n**, **o** In P6 lateral wall, differentiating ependymal cells with few cilia are MN1-positive (arrows). A later differentiating multiciliated ependymal cell is MN1-negative (arrowhead). Experiments were performed twice with equivalent results.

MN1-immunoreactivity was detected in the nucleus and cytoplasm of nascent immature ependymal cells of the mouse pup ventricular zone (Fig. 6e, g, l–o). These MN1-positive ependymal precursors are in an intermediate stage of differentiation, referred to as the *individualized stage*, and exhibit centrioles and few to multiple short cilia. This stage occurs between the earliest stage of ependymal differentiation, i.e., the *halo stage*, involving generation of multiple pro-centrioles, and fully mature ependyma demonstrating mature centrioles and long motile cilia^51^ (Fig. 6). Cytoplasmic staining for IGF2 was also observed in these cells (Fig. 6b, h–k), suggesting that *MN1-BEND2* or other ependymal tumors may originate from similar immature ependymal cells derived from vRG^9,52^. IP cells basal to the OSVZ also stained for MN1, but not IGF2. Nevertheless, such cells could also theoretically give rise to *MN1-BEND2* tumors.

### MN1-BEND2 tumors show increased expression of imprinted genes

**Fig. 7:**
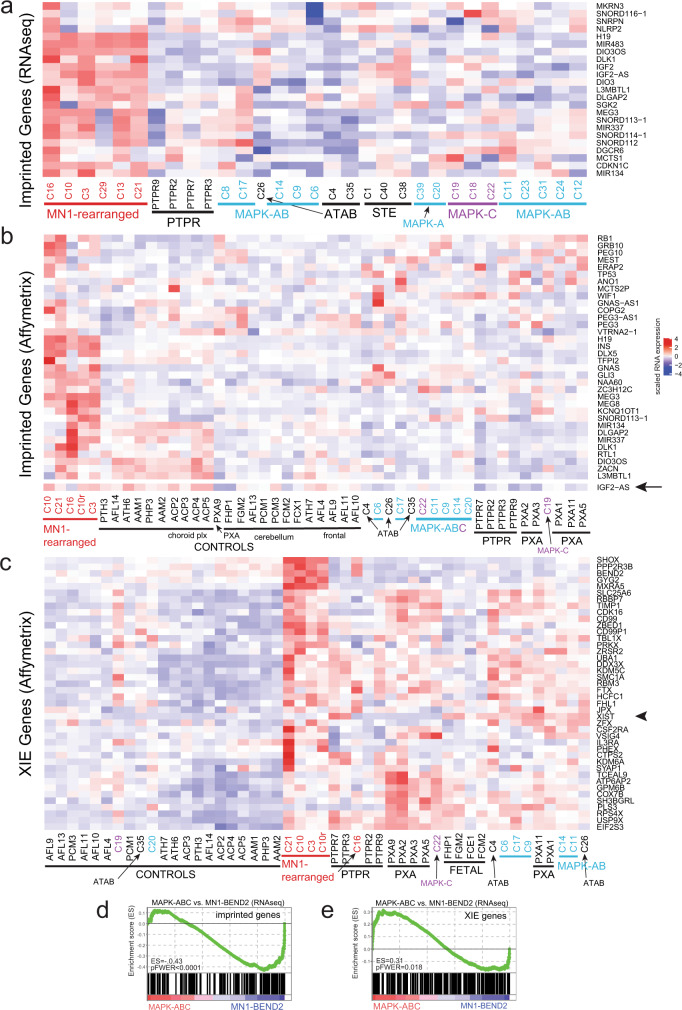
*MN1-BEND2* AB-like tumors exhibit relative overexpression of imprinted genes while MAPK-ABC tumors show relative overexpression of X-inactivation escape genes. **a** Heatmap of unsupervised hierarchical clustering of RNAseq expression data for a subset of imprinted genes. **b** Similar data from the Affymetrix analysis including controls and PXA. **c** Heatmaps for expression of subsets of XIE genes from the Affymetrix data. *IGF2* is absent in the NCBI annotation for the HTA 2.0 array as of April 2021. *IGF2-AS* (arrow) may serve as a surrogate marker for *IGF2* and was upregulated in *MN1*-rearranged tumors and normal choroid plexus (choroid plxs). The latter develops as an out-pouching of the ependyma and is known to express *IGF2*^128^. The *XIST* gene message is indicated by the arrowhead. *MN1*-rearranged tumors are highlighted in red, MAPK-AB in blue, and MAPK-C in purple. Complete source data for heatmaps are available as Supplementary Fig. 8-9 and 13. **d** GSEA plot showing enrichment of imprinted gene expression in *MN1-BEND2* versus MAPK-ABC tumors. **e** GSEA plot depicting enrichment of XIE gene expression in MAPK-ABC versus *MN1-BEND2* tumors. GSEA is two-sided with adjustment for multiple comparisons. Tumors or control tissues comparatively enriched for neural stem cell gene sets are depicted in larger and bold font. Colors are arbitrary.

Unsupervised hierarchical clustering of 139 currently annotated imprinted genes culled from the literature^53–55^ (Supplementary Data 6), revealed overexpression of distinct signature blocks of imprinted genes in *MN1*-rearranged tumors as compared to MAPK-ABC lesions (Fig. 7a, b and Supplementary Figs. 8, 9). Examples include genes from the IGF2-H19 imprinted locus on chromosome 11p15, e.g., *IGF2*, *H19*, and *miR483*, and *DLK1*, *DIO3,* and *miR134*, which reside within the DLK1-DIO3 imprinted locus at 14q32 and whose misregulation is linked to schizophrenia and bipolar disorder^56,57^. miR483 was also found to be highly expressed in *MN1-BEND2* tumors and RELA STE compared to non-*MN1-BEND2* AB-like tumors independently by Nanostring methodology (Supplementary Fig. 10). *GNAS*, an imprinted NEC gene encoding a G-protein α-subunit activating adenylate cyclase, was also relatively overexpressed in *MN1-BEND2* and some other AB-like tumors, but only in female patients (Supplementary Figs. 7, 8). Most of these genes were also upregulated in *MN1*-rearranged tumors compared to normal and fetal brain (Fig. 7b and Supplementary Fig. 9).

GSEA revealed that imprinted gene transcripts are significantly enriched in *MN1-BEND2* compared to MAPK-ABC tumors (ES = |0.43| , pFWER < 0.0001) (Fig. 7d) and all other tumor types and controls (pFWER ≤ 0.0001) (Supplementary Data 7).

### Fusions involving imprinted and chromosome X genes occur predominantly in AB-like tumors from female patients

Fusions containing the imprinted gene maternally expressed 8 (*MEG8*), encoding a small nucleolar RNA, were only present in tumors from females, in four MAPK-AB lesions and four of five *MN1-BEND2* tumors (Fig. 4f, Supplementary Fig. 11A, Supplementary Data 2). Most were *SNHG23-MEG8* (chromosome 14:14) fusions, with the exception of *SNORD114-26-MEG8* (14:14) in two *BRAF*^*V600E*^-mutant cases and *RUNX1-MEG8* (21:14) in one *MN1-BEND2* case. *SNORD114* resides on chromosome 14 within the same imprinted region as *MEG8*. All were out-of-frame rearrangements demonstrated by multiple reads. These fusions correlate with chromosome 14 instability previously observed in *MN1*-rearranged tumors^4^. Fusions containing *GNAS* (at 20q13) also only occurred in female cases (Supplementary Fig. 11A, Supplementary Data 2).

Fusions involving imprinted genes overall occurred at similar frequency in *MN1-BEND2* (2.6/case, *n* = 5) and MAPK-ABC tumors (2.1/case, *n* = 15), but were almost twofold more frequent in *BRAF*^*V600E*^ cases (4.2/case, *n* = 6), and approximately tenfold more frequent in females (2.7/case, *n* = 16) compared to males (0.25/case, *n* = 4). Mutations in imprinted genes were found at similar frequency in *MNI-BEND2*, *BRAF*^*V600E*^, and MAPK-ABC tumors (1.0, 0.7, and 1.7/case, respectively), and were most common in males (2.5/case).

Considering all genes in all AB-like tumors, fusions were 4.9-fold more frequent in female versus male patient tumors (Table 2). Fusions involving chromosome X were 5.3-times more common in tumors from females (*P* = 0.017) and occurred almost entirely in *MN1-BEND2* and *BRAF*^*V600E*^-mutant tumors. Fusions involving chromosome 22 were 17-fold more common in female patients (*P* = 0.0035, Table 2). *Statistically significant* p* < 0.05.

**Table 2:** Total chromosome fusions for all chromosomes, chromosome X and chromosome 22 for female and male patients.

	Total Fusions	Multiple Reads	# Cases
**All Chromosome Fusions**			
FEMALE			
Total Count	2738	1488	20
Per Case	136.9	74.4	
MALE			
Total Count	221	107	7
Per Case	31.6	15.3	
F:M Ratio	4.87		
*p* value*	0.0061		
**Chromosome X Fusions**			
FEMALE			
Total Count	119	61	20
Per Case	5.95	3.05	
MALE			
Total Count	9	4	7
Per Case	1.3	0.6	
F:M Ratio	5.34		
*p* value*	0.017		
**Chromosome 22 Fusions**			
FEMALE			
Total Count	78	49	20
Per Case	3.9	2.5	
MALE			
Total Count	3	1	7
Per Case	0.4	0.1	
F:M Ratio	17.15		
*p* value*	0.0035		

*Statistically significant p < 0.05.

### X-inactivation escape genes are more widely expressed by and mutant in MAPK supercluster AB-like tumors

We also compiled a list of 95 XIE genes and 105 genes exhibiting variable XIE status (Supplementary Data 6)^56^.

Overall, MAPK-ABC and other non-*MN1-BEND2* AB-like tumors showed overexpression of more XIE genes than *MN1-BEND2* tumors (Fig. 7c, Supplementary Figs. 12, 13). *XIST*, an XIE gene encoding a long noncoding RNA that regulates X-inactivation on the inactive X-chromosome^58^, was relatively overexpressed in MAPK-ABC ABs from female patients compared to other tumors and controls (Supplementary Figs. 1–3, 12, 13). Indeed, GSEA showed that XIE gene expression is significantly enriched in MAPK-ABC tumors versus *MN1*-rearranged tumors (ES = |0.31|, pFWER = 0.018) (Fig. 7e).

Three *BRAF*^*V600E*^-mutant MAPK tumors additionally contained fusions with *XIST* as the downstream gene (Supplementary Data 2). Mutations in XIE genes *USP9X, NLGN4x*, *PLXNB3* (involved in axonal guidance and glioma invasion), *SYAP1*, *SRPX2,* and *ZFX* (*ZFX-SMARCA1* fusion) occurred in non-*MN1-BEND2*, predominantly female cases. Some XIE genes were also overexpressed or mutant in *MN1*-*BEND2* tumors, e.g., *SHOX* and *ZFX*.

Deleterious SNPs and other non-fusion mutations of XIE genes occurred slightly more frequently in MAPK-ABC tumors (5.0/case) and the *BRAF*^*V600E*^-mutant subgroup (5.3/case) compared to *MN1*-*BEND2* cases (4.0/case), and in females (5.9/case) compared to males (3.0/case) (Supplementary Fig. 11B, Supplementary Data 2). However, fusions involving XIE genes occurred 3.6-fold more frequent in *MN1-BEND2* tumors (4.0/case) than MAPK-ABC tumors (1.2/case) and 4.6-times as frequent in females compared to males (2.3 vs. 0.5/case, respectively).

### Expression of AB-like tumor genes correlate with gene promoter methylation

In order to confirm that differential expression of AB-like tumor genes can be, in some cases, attributable to differential promoter methylation, we integrated RNAseq expression and DNA methylation data using the mCSEA R package^59^, which correlates differentially methylated regions (DMRs) with expression of nearby downstream genes. The expression of several *MN1-BEND2* overexpressed genes within the IGF2-H19 and DIO3-DLK1 imprinted regions and other *MN1-BEND2* and MAPK-ABC overexpressed genes significantly correlated with their promoter methylation status (Supplementary Fig. 14).

## Discussion

MN1 coregulates transcription and chromatin remodeling. Its C-terminal truncation is associated with a distinct craniofacial/brain malformation syndrome^60,61^. MN1 is implicated as a tumor suppressor in meningioma and an oncoprotein in acute myeloid leukemia^62–64^. Alteration of these potential functions could be important in *MN1-BEND2* and other *MN1*-rearranged tumors, e.g., containing *MN1-CXXC5* fusions^6,65^.

BEND2’s function is unknown however BEN domain-containing homologs participate in chromatin remodeling and transcriptional repression^66,67^. As a putative chromatin remodeling protein, BEND2 dysregulation could potentially influence gene expression. We identified *MN1-BEND2* fusion transcripts in five tumors from females, all over-expressing *MN1* and *BEND2* mRNA. *BEND2* overexpression has also been documented in an additional *MN1-BEND2* tumor^68^. An *MN1*-rearranged tumor from a male patient in our series, lacking *MN1-BEND2* (or other *MN1* or *BEND2* fusions), did not overexpress *MN1*, but did overexpress *BEND2*. It clustered with *MN1-BEND2* tumors by RNA expression, but not DNA methylation.

Of eight recently reported AB-like tumors demonstrating *EWSR1*-*BEND2* fusions (Supplementary Data 8), one had documented high *BEND2* expression^69^. Most were infratentorial, involved the medulla or spinal cord and some clustered near HGNET-MN1 by tSNE analysis of DNA methylation^70^. All but one in these anatomic regions occurred in males. Two frontal lobe cases were in females. *EWSR1* is located at chromosome 22q12.2 near *MN1*, thus both translocations involve the same 22q12 general breakpoint region. *BEND2* overexpression may thus be significant in the tumorigenesis of *MN1*-*BEND2* and similar tumors. Indeed, *BEND2* appears to be normally expressed in the brain only at very low levels (Fig. 5).

**Fig. 8:**
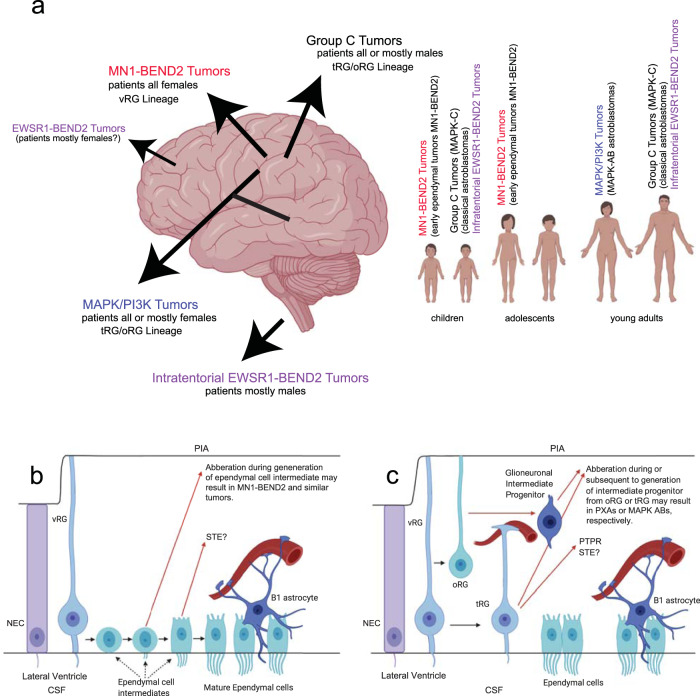
Astroblastoma-like tumors show neural stem cell type-specific transcriptional lineage of developmentaly early (vRG) to later (oRG and tRG) radial glia and clinically present in comensurate sequencial patient ages. **a** Summary of anatomic locations and patient age groups of molecular subtypes of AB-like tumors. **b** Hypothetical model for EET MN1-BEND2 tumorigenesis. **c** Hypothetical model for MAPK-ABC astroblastoma tumorigenesis. GSEA and RNA expression patterns of *FOXJ1* and *IGF2* suggest a temporal progression of early to late vRG to tRG and oRG genes and development of related tumor types: EET MN1-BEND2, *FOXJ1*+/*IGF2*+; RELA STE, *FOXJ1*+/*IGF2*+; PTPR, *FOXJ1*+/*IGF2*−; MAPK-ABC and PXA, *FOXJ1*−/*IGF2*.

All reported tumors with confirmed *MN1-BEND2*, and available demographic data, occurred in females aged 4–18 years (mean 10.4 yr, *n* = 13) (Supplementary Data 8). Tumors occurred in the frontal, parietal or occipital lobe, *predominantly the parietal lobe*, but not the temporal lobe or infratentorial as seen in non-*MN1-BEND2* AB-like tumors (Fig. 8a, Table 1, Supplementary Data 8). In contrast, the mean age of presentation of *BRAF*^*V600E*^-mutant AB tumors in our series was 24.7 years (*n* = 6), and 29.1 years for MAPK-ABC cases (*n* = 15). It, therefore, appears that AB-like tumors related to earlier developmental stages of NSCs (RG), i.e., *MN1-BEND2* tumors, clinically present earlier in life compared to tumors transcriptionally related to later RG, e.g., MAPK-ABC ABs and PTPR^17^.

The transition of embryonic stem cells to NSCs is accompanied by changes in DNA methylation. Additional methylation changes allow expression of glia-specific genes during the mid-gestational shift from primarily neurogenesis to gliogenesis^28,71,72^. Further epigenetic changes occur during increased transcriptional activity and synaptic remodeling in childhood through young adulthood^73,74^. DNA methylation and gene expression patterns in *MN1-BEND2* and MAPK-ABC tumors may reflect these developmental dynamics and partially explain their different ages of clinical presentation. Dysregulation or mutation of chromatin remodeling genes, e.g., *MN1*, *BEND2*, *ATRX*, *CXXC5*, *CREBBP,* and *SMARCAD1*; histone modifier genes, e.g., *CHD3*, *KMT2C*; and/or possibly X-inactivation effectors could alter DNA methylation and potentially contribute to AB tumorigenesis.

The terms *early-*, *mid-*, and *late-RG* have been used in model systems^28,75^. Ziller et al.^28^ defined mid-RG as corresponding to peak neurogenesis and early gliogenesis, and late-RG to mostly gliogenesis. Like vRG, early-RG expressed *PAX6* and *FOXJ1*. Late-RG highly expressed *OLIG2* and *TCF4*, similar to oRG and MAPK-ABC ABs.

*MN1-BEND2* tumors exhibit high expression of several NEC and vRG markers and low expression of the oRG markers *FAM107A*, *LIFR,* and *HOPX* (Supplementary Fig. 5A, B, D). Unlike *MN1-BEND2* tumors, MAPK-ABC tumors and tRG are enriched for *CRYAB*^12^ (Supplementary Fig. 5C). The latter is more highly expressed after 25 pcw, consistent with tRG expressing a later transcriptional program than vRG (Fig. 5).

Our data suggest that *MN1-BEND2* tumors ultimately originate from early vRG-derived ependymal progenitors or a residual population of related cells. Such cells may become neoplastic due to epigenetic and/or genetic events in utero or during the dramatic growth of the brain during childhood. The correlation of *MN1-BEND2* tumor transcriptomes with early NSC types (NEC and vRG) and MAPK-ABC tumor, PTPR, and PXA transcriptomes with later NSC types (tRG and oRG) suggests that mutagenic events in early brain development may manifest as tumors in both childhood and adulthood, or that some RG or RG-derived progenitors persist beyond adolescence.

NEC and vRG project an apical primary cilium into the lateral ventricle, facilitating IGF2 binding from the CSF^76^. Unlike adult rodent lateral ventricle NSCs (B1 astrocytes), NSCs in the adult rodent dentate gyrus express IGF2, which is required for their proliferation in an apparent autocrine fashion^73,77^. This regional difference in *IGF2* expression was attributed to differential imprinting^73^. We observed IGF2 immunoreactivity in immature ependymal cells of the fetal mouse lateral ventricular zone. Thus, IGF2 produced by differentiating ependymal cells may possibly be a critical pro-proliferative signal in the fetal lateral ventricular zone. This seems plausible considering the requirement of the ependymal layer by B1 astrocytes^78^. Alternatively, this additional IGF2 may be needed by the developing ependyma themselves or other nearby cells.

MAPK supercluster tumors demonstrated high expression, mutation, and fusion of genes reflecting glial and neural differentiation. GSEA supports that these tumors express transcriptomes reflective of tRG and oRG. PXA was more highly enriched for oRG genes compared to tRG genes. The mixed pattern of tRG and oRG gene expression by MAPK-ABC and PXA might be explained by the possibility that gene expression of late RG types and/or their progeny IPs might vary with development over time, and that the published tRG and oRG gene sets represent developmental timeline-dependent snapshots.

MAPK-ABC tumors exhibit overexpression and mutation of genes involved in neurocognitive disorders characteristic of children and young adults. *TCF4* is implicated in schizophrenia and Pitt-Hopkins autism-like syndrome^79,80^. *ANK3* has been associated with autism, bipolar disorder, and schizophrenia^81^. *KALRN*, *TRIO,* and *DISC1* misexpression or mutation are implicated in attention deficit hyperactivity disorder, autism, bipolar disorder, schizophrenia, and other conditions^82–85^. KALRN protein (*kalirin*) isoforms show increased expression in adolescence and early adulthood when schizophrenia and many MAPK supercluster AB-like tumors occur^86,87^.

Group C and other non-*MN1-BEND2*/*non-BRAF*^*V600E*^-mutant AB-like tumors exhibit *AHNAK* and *SYNE1* mutations. Although *AHNAK* has been characterized as a tumor suppressor^88^, *AHNAK* and *SYNE1* have also been implicated as cancer genes^89^. AHNAK is necessary for pseudopod formation, a key step in the epithelial to mesenchymal transition and tumor cell invasion^44^. *SYNE1* encodes spectrin repeat-containing nuclear envelope family member 1, which is involved in nucleolemmal cytoskeletal anchoring^23,90^.

An intriguing aspect of *MN1-BEND2* and MAPK-AB tumors is their striking female predominance of up to 9:1 or greater^4^. We previously observed chromosome X gains in seven AB-like tumors from female patients and only one in a male patient; X loss was seen in four tumors from females and none from males^4^. Here, fusions involving chromosome X were over five times more common in female patient tumors, and almost entirely in *MN1-BEND2* and *BRAF*^*V600E*^-mutant tumors. Chromosome 22 fusions were even more frequent in tumors from females. Meiotic recombination of chromosome 22 is greater in females than males^91^. How this relates to non-homologous mitotic recombination is unclear.

High expression of imprinted genes in *MN1-BEND2* tumors and XIE genes in MAPK-ABC tumors suggest a potential role for differential DNA methylation in driving their tumorigenesis. *BEND2* itself exhibits variable XIE status^15^. XIE genes can be activated by epigenetic changes in chromatin structure and are frequently mutated in cancer, especially female predominant cancers^92,93^.

The imprinted homeobox gene *DLX5* may escape imprinting and be biallelically expressed in mice and patients with the nearly entirely female disease Rett syndrome^94,95^. The latter is characterized by mutations in chromosome X gene *MECP2* encoding methyl-CpG binding protein 2, which binds methylated DNA and suppresses transcription. We found frequent overexpression of imprinted genes *DIO3*, *IGF2, H19*, and others, correlating with decreased methylation of their promoters in *MN1-BEND2* tumors. Our data suggest that epigenetic and genetic alterations associated with AB-like tumors possibly involves loss of imprinting, leading to increased expression and sometimes subsequent mutation of affected genes^96,97^. Loss of imprinting is linked to several cancers and pediatric syndromes, *e.g*., Beckwith-Wiedemann syndrome involving alteration of the IGF2 imprinting control region and increased risk for Wilms tumor and other neoplasms^98^. High expression of imprinted genes in *MN1-BEND2* tumors could alternatively be due to retention of fetal methylation patterns. Interestingly, DNA methylation and expression of imprinted genes in the brain is not only developmentally time dependent, but is also anatomic region dependent^99–101^, which may possibly relate to anatomic locations in which AB-like tumors occur (Fig. 8a).

We provide multiple lines of evidence that *MN1-BEND2* and MAPK-ABC *astroblastomas* are of radial glia lineage and likely derived from vRG, and oRG and/or tRG or their close progeny, respectively (Fig. 8b, c). This interpretation is limited however by the mostly correlative and non-mechanistic nature of the data. For instance, the mostly adult glioma, glioblastoma, is thought to originate from subventricular zone B1 astrocytes^102^. We cannot completely rule out that some AB-like tumors may be derived from B1 astrocytes, as the transcriptome of B1 astrocytes is largely undefined.

PTPR has been hypothesized to be derived from the subcomissural organ (SCO) located in the third ventricle. GSEA data strongly suggest that PTPR are closely related to tRG and thus derived from them. tRG resemble an incompletely defined RG-like ventricular zone “ependymoglial” cell known as the *tanycyte*, which may also project a single cilium into the ventricle and an elongated basal process to a subependymal capillary^103^. Such cells are notably found in various third ventricle locations including the SCO. Tanycytes may thus represent persistent tRG capable of generating PTPR. Notably, PTPR strongly express PAX6, which is expressed by tRG^12^. It has also been proposed that ABs may be derived from tanycytes^104^, potentially consistent with our findings of partial enrichment of tRG genes in MAPK-ABC tumors.

Recently, tumor stem cells expressing the oRG marker *PTPRZ1* capable of MST were found in glioblastoma^105^. This supports the possibility that residual oRG or oRG-derived IP cells persist into adulthood and lead to tumor formation. Invasive properties of these oRG-like tumor stem cells^105^ may explain why oRG-related MAPK supercluster ABs have a poorer prognosis than *MN1-BEND2* tumors^4^. It may also be that B1 astrocytes or other glioblastoma initiating cells can differentiate to attain oRG-like properties. The relevance of either possibility in adult gliomagenesis remains to be determined.

Because *MN1-BEND2* containing AB-like tumors are generally associated with excellent patient survival^4^, they should not be termed high-grade neuroepithelial tumors^6^. Because *MN1-BEND2* tumors are most likely derived from cells destined to become ependymal cells, they also should not be called astroblastomas, but rather early ependymal tumors with *MN1-BEND2* fusion (EET MN1-BEND2). Of practical clinical diagnostic importance, we show that *MN1-BEND2* tumors can be differentiated from other AB-like tumors by their strong diffuse MN1 nuclear immunoreactivity and absent diffuse GFAP staining. We recommend that MN1 and GFAP immunohistochemistry, DNA methylation analysis, and confirmation of the *MN1-BEND2* fusion (and if negative, *MN1-CXXC5* or *EWSR1-BEND2 fusions*) by FISH, RT-PCR, or sequencing comprise an integrated approach to the diagnosis of EET MN1-BEND2 and other AB-like tumors.

As oRG, and likely tRG, give rise to IPs destined to become neurons or *glia*, the coinage of “astroblastoma” by Bailey and Cushing^106^, indicating derivation from an astrocyte progenitor, was amazingly insightful. We propose that the term *astroblastoma* be reserved for MAPK supercluster AB-like lesions, particularly DNA methylation Group C tumors, which may exhibit classic astroblastoma histomorphology.

Lastly, our data identify the IGF2 pathway and the multidrug transporter ABCC1 as candidate therapeutic targets in EET MN1-BEND2^107,108^. While BRAF and downstream MEK and/or PI3K/AKT/mTOR pathway inhibitors may be useful in treating MAPK-ABC astroblastomas^109,110^.

## Methods

### Tumors

Construction of our clinical cohort of histologically-defined AB-like cases has been described^2^. The cohort was augmented with three additional AB cases with available material for further study (C39, 43–44) and an additional five tumors with AB-like histological features, for a total of 35 AB-like cases in the current cohort. All control brain tissue was retrospectively obtained from archived material from routine hospital autopsies. Equivalent fetal age control brain tissue was obtained from autopsies of spontaneous abortuses or deceased premature infants at Nationwide Children’s Hospital, Columbus, Ohio. Autopsies were requested by patient’s families or their physicians for medical reasons and performed with the consent of the patient’s next of kin, completely independent of the studies described herein. Informed consent for the use of archival biopsy or autopsy tissue was not obtained, nor required by the institutional review board. No patients or patient families were compensated for these studies. Institutional review board approval from the University of Louisville was obtained for use of control and case material (Approval number 17.0984). *MN1*-rearrangement was determined by FISH (cases C3, C7, C10, C13, C16, C21, and C29^5^) and confirmed by CNV analysis using the Illumina EPIC BeadChip 850K microarray data from these and two additional samples (C43 and C44).

### DNA methylation

Genomic DNA methylation analysis was performed on an additional fourteen tumors (C8, C15-18, C21, C27, C31, C35-36, C41-44) using the Illumina EPIC BeadChip 850K microarray similar to as previously described^6,111,112^. Genomic DNA was extracted from 4 μm sections of formalin-fixed paraffin-embedded (FFPE) tissues using the AllPrep DNA/RNA FFPE Kit (Qiagen, Hilden, Germany) and quantified using a Qubit High Sensitivity assay (Thermo Fisher Scientific, UK), before bisulfite conversion of up to 250 ng DNA, using the EZ DNA Methylation kit (Zymo Research, Irvine, CA). Bisulphite-converted FFPE DNA was processed with the Infinium FFPE DNA Restore kit (Illumina, UK) and assayed on the Infinium Methylation EPIC BeadChip (Illumina), according to the Infinium HD FFPE Methylation Assay automated protocol (Illumina).

The tSNE plot was generated using the 32,000 most variably methylated probes of the study samples and 2801 German Cancer Center (DKFZ) reference samples as previously described^111^. The later consists of 2729 tumors (comprising 76 pathological diagnoses) and 72 normal brain tissues.

### RNA isolation

Total RNA was extracted from three 30 μm scrolls cut from FFPE tissue blocks containing 90% tumor or more, using the RecoverAll total nucleic acid isolation kit (Ambion/Thermo Fisher Scientific, Waltham, MA) per the manufacturer’s instructions. RNA was then passed through concentration and clean-up columns (Norgen Biotek, Thorold, ON) and quantified using a Nanodrop spectrophotometer (Thermo Fisher Scientific, Waltham, MA).

### RNAseq

Capture-based RNAseq was performed using a modified SureSelect strand-specific RNA library prep and target enrichment protocol (Agilent Technologies, Santa Clara, CA). Briefly, 200 ng of FFPE-derived total RNA was heated to 65 °C for 5 min and used for first-strand cDNA synthesis. Second-strand cDNA was synthesized, and the ends repaired. The 3ʹ ends of ds-cDNA were adenylated, ligated with adapters and fragments were PCR-amplified. PCR product (700 ng) was hybridized overnight to SureSelect XT Human All Exon V5 + lncRNA baits. The hybridized fragments were captured on magnetic beads and PCR-amplified using uniquely barcoded index primers. Barcoded libraries were pooled and underwent paired-end 150 bp sequencing on an Illumina HiSeq4000 instrument (Illumina, San Diego, CA).

For gene expression, read alignment was performed with hisat2-2.0.5^113^ to human genome GRCh38 and aligned files were converted to bam and sorted with samtools v1.3.1^114^. Alignment QC was generated with RSeQC v2.6.4^115^. Counts from the subread package v1.5.1^116^ were used to count the number of reads aligning to Ensembl IDs according to GRCh38.86 annotation.

Fusion detection was performed using JAFFA^117^ after merging across replicate lanes. Variant calling was performed following GATK’s best practices for variant calling in RNAseq using a 2-pass STAR alignment. Alignments were made with STAR v2.5.2a^118^ to human reference genome GRCh38. The junctions detected in all samples were merged and used to make a custom STAR genome index for a second pass alignment. Picard v2.4.1 was used to preprocess the alignment files, add read groups, mark duplicates, sort, and index. GATK v3.6^119^ was used to split reads containing junctions, realign around indels, and recalibrate the base qualities. Samtools v1.3.1 was used to generate mpilup format. BRAF mutations were identified by a visual inspection of the alignment BAM in the Integrated Genomic Viewer^120^ since they were not detected by VarScan analysis. VarScan v2.4.1^121^ was used to call and filter variants from mpilup format from the whole genome. Called variants were annotated with Ensembl Variant Effect Predictor (VEP)^122^. Variants were eliminated if listed in dbSNP alone. Variants with MEDIUM or HIGH impact as designated by VEP were retained. After filtering variants detected by the same set of reads were eliminated. PolyPhen, SIFT, and FathmmMKL predictions were determined using respective versions available July 2018.

### cDNA microarrays

Affymetrix GeneChip Human Transcriptome Array 2.0 (HTA-2) microarray analysis was performed according to the manufacturer’s instructions using the GeneChip WT Pico and Sensation Plus sample preparation kits (Thermo Fisher Scientific). In brief, 50 ng of total RNA was converted to single-stranded cDNA with T7 promoter sequence at the 5′ end. A 3′ Adaptor, as a template for double-stranded cDNA synthesis, was added and the single-stranded cDNA was converted to double-stranded cDNA. Six amplification cycles were applied as recommended by the protocol to enrich the samples. The amplified product was converted to cRNA and purified using purification binding beads. Eluted cRNA yield was measured by NanoDrop. cRNA (20 μg) were reverse transcribed to sense-strand cDNA containing dUTP at a fixed ratio relative to dTTP. The cRNA was removed using RNase H and the 2nd-cycle single-stranded cDNA was purified using purification binding beads. The eluted single-stranded cDNA yield was determined using the NanoDrop and 5.5 μg was fragmented by uracil-DNA glycosylase and apurinic/apyrimidinic endonuclease 1 (APE 1). The fragmented ss-cDNA was labeled by terminal deoxynucleotidyl transferase, and then used to prepare the Hybridization Cocktail, injected and hybridized in the array for 16 h at 45 °C in an oven with rotation at 60 rpm. After overnight hybridization, the hybridization cocktail mix was extracted from the array. Each array was washed and stained using Affymetrix GeneChip Fluidics Station 450 according to the user manual (P/N 08-0295) and the arrays were scanned using GeneChip Scanner 7G. Each array image was collected using the GeneChip Command Console, QC, saved as.cel files and saved for further analysis.

### RNA expression analysis, dimensionality reduction, and clustering

For the RNAseq data, transcripts with 0 counts > 50% were removed. Data were normalized with Limma-voom R package with quantile normalization^123^. The Ensembl IDs were mapped to unique gene symbols using the Bioconductor org.Hs.eg.db package (ver: 3.13.0) using median summarization.

For the microarray data, probe-level data were generated by analyzing the raw signal intensities from the raw Affymetrix CEL data files for each probe set using the *rma* algorithm for background intensity correction and normalization in the oligo package (ver: 1.56). Batch effect related to the sample preparation kit was reduced using the combat function of the sva package (ver: 3.4). The probes were mapped to unique gene symbols using the hta20transcriptcluster.db package (ver: 8.8) with median summarization.

Differential gene expression between groups of samples in RNAseq and Affymetrix datasets was performed using analysis of variance thresholding with a two-sided p-value to 0.05. Expression values of the genes differentially expressed in both datasets were scaled and denoted on a heatmap with the respective samples. Heatmaps in Figs. 1, 5, 8, S1–S3, S7, S8, and S11, S12 were generated by unsupervised hierarchical clustering.

Dimensionality reduction of the expression data with t-Distributed Stochastic Neighbor Embedding (tSNE) was performed to facilitate visualization using the following parameters (dimensions = 2, perplexity = 4, iterations = 5000) using the Rtsne package (ver: 0.15). All analyses were performed using R software (ver: 4.1).

### Gene set enrichment analysis (GSEA)

GSEA was performed using GSEA software from the Broad Institute, Cambridge, MA (ver: 4.1)^124,125^ using the following settings (permutations: 1000; permutation type: gene_set; enrichment statistic: weighted; metric for ranking genes: *t* Test).

### Differential gene promoter methylation and expression correlation

Raw methylation files were read and normalized using the sesame package (ver: 1.10.4) with the default settings. Differentially methylated gene promoters were identified using the mCSEA package (ver: 1.12). Correlation between methylation of gene promoters and expression levels was quantified using the mCSEAIntegrate function. This correlates differentially methylated regions (DMRs) with the expression of nearby downstream genes^59^.

### Nanostring MicroRNA

MicroRNA (miR) profiling of tumor samples was performed using the Nanostring nCounter system (Nanostring Technologies, Seattle, WA)^126^. One hundred nanograms of total RNA was annealed with multiplexed DNA tags (miR-tags) complementary to specific target miR sequences. Mature miRs were bound to specific miR-tags using a ligase and excess tags were enzymatically removed. Tagged miRs were diluted 1–5, and 5 µl was combined with 5 µl of capture probes and 20 µl of reporter probes in hybridization buffer. Probes were allowed to complex with specific target sequences overnight (16–20 h) at 65 °C. Excess probe was removed using a two-step magnetic bead-based purification (nCounter Prep Station) and target/probe complexes were immobilized onto cartridges. Reporter probe counts were obtained for each sample using the Gen1 nCounter Digital Analyzer, which utilizes a CCD camera-equipped microscope to image the immobilized fluorescent reporters via a high-density scan encompassing 600 fields of view. Raw data output was imported into nSolver for normalization (http://www.nanostring.com/products/nSolver). For analysis, data are the geometric mean normalized and then log10. For heatmap generation, data were scaled and truncated as follows. For scaling, the data are mean centered and divided by the SD of the miR. Truncation was done at a value of 4.0 to adjust for rare very large outlier values in the matrix.

### Immunohistochemistry

Immunohistochemistry was done using 4 µ thick sections of FPPE tissue. Immunohistochemical stains for GFAP clone EP672Y (Cell Marque 258R-16, lot 32653, 1:25), MN1 (Proteintech 24697-1-AP, lot 00021048, 1:40) and IGF2 (Abcam; ab9574, lots GR31975-61, and GR31975-64, 1:500) were performed on the Leica Bond III using Leica Epitope Retrieval 1 (Leica; AR9961) for GFAP, and Epitope Retrieval 2 (Leica; AR9640) for MN1 and IGF2. Retrieval was performed for 20 min, followed by 15 min primary antibody incubation, and Bond Polymer Refine Detection (Leica; DS9800). Hematoxylin was used as a counterstain. Positive IGF2 staining was defined as dark granular perinuclear cytoplasmic staining.

### Western blotting

Protein lysates of frozen tumor tissue were prepared as previously described^127^, adding to the lysis buffer 1 mM sodium orthovanadate and 5 mM sodium fluoride. Fifteen micrograms of protein were resolved on 10% bis-tris gels (Invitrogen) and transferred to PVDF membranes. Primary antibodies, BEND2 (Invitrogen PA5-31747, lot UA2709054B, 1:500), IGF2 (Abcam ab262713, lot GR3295940-1, 1:500), MAP3K5 (Millipore MABC632, lot VP1812286, 1:1000), ABCC1 (Abcam ab24102, lot GR3247402-6, 1:50), FOXJ1 (Novus NBP1-87928, lot H119212, 1:500), MN1 (Invitrogen PA5-38666, lot UD2755192, 1:500), TCF4 (Proteintech 22337-1-AP, lot 00050018, 1:500), and β-actin (Sigma A2228, lot 085M4754V, 1:10,000), were incubated for 90 min at room temperature, followed by a 60 min incubation with HRP-conjugated secondary antibodies (Cell Signaling, 1:2000). Uncropped and unprocessed scans of the most important blots are supplied in the Source Data file.

### Animals

Animal studies were performed in accordance with the guidelines of the European Community and French Ministry of Agriculture and were approved by the “Direction départementale de la protection des populations - Paris (authorization 9343201702211706561). Mice used in this study are Centrin2-GFP (CB6-Tg(CAG-EGFP/CETN2)3- 4Jgg/J; The Jackson Laboratory). Animals were group-housed with free access to food and water in controlled temperature conditions (room temperature controlled at 21–22 °C, humidity between 40 and 50%), and exposed to a conventional 12-h light/dark cycle. Experiments were performed on embryos at embryonic (E) days E16, and neonate pups at postnatal day 0–10 (P0–10) of both sexes. All procedures were approved by the Ethical Committee CEEA-005 Charles Darwin (authorization 9343-201702211706561) and conducted in accordance with EU Directive 2010/63/EU. All efforts were made to reduce animal suffering and minimize the number of animals, in compliance with all relevant ethical regulations for animal testing and research.

### Immunofluorescence

Animal brains were dissected out in cold PBS from embryos or newborn animals euthanized by decapitation. Lateral ventricle whole-mounts were prepared as previously described^9^. Whole-mount brain sections were fixed for 30 min in 4% PFA, incubated for 1 h in blocking solution (1X PBS with 0.1% Triton X-100 and 10% fetal bovine serum) at room temperature and were incubated overnight at 4 °C in primary antibodies diluted in blocking solution. The primary antibodies used targeted polyglutamylated tubulin (Adipogen AG-20B-0020-C100, 1:1000), MN1 (Proteintech 24697-1-AP, lot 00021048,1:100), or IGF-2 (Abcam ab9574, 1:100). The following day, they were stained with species-specific AlexaFluor fluorophore-conjugated secondary antibodies for 2 h at room temperature (1:400, Thermo Fischer Scientific or Jackson ImmunoResearch Labs). Finally, the whole-mounts were mounted with Fluoromount-G mounting medium (Southern Biotech, 0100-01).

### Reporting summary

Further information on research design is available in the Nature Research Reporting Summary linked to this article.

## Supplementary information

Supplementary Information

Description of Additional Supplementary Files

Supplementary Data 1

Supplementary Data 2

Supplementary Data 3

Supplementary Data 4

Supplementary Data 5

Supplementary Data 6

Supplementary Data 7

Supplementary Data 8

Reporting Summary

## Data Availability

The raw and processed RNAseq, Affymetrix, MethylationEpic BeadChip, and Nanostring microRNA data generated in this study are deposited at the public repository Gene Expression Omnibus (GEO) under the accession numbers GSE165351, GSE165813, GSE166569, and GSE196697, respectively. The remaining data are available within the Article, Source Data File, Supplementary Data, and Supplementary Information. Source data are provided with this paper.

## Source data

Source Data
