# Author Correction: Astroblastomas exhibit radial glia stem cell lineages and differential expression of imprinted and X-inactivation escape genes

**DOI:** 10.1038/s41467-023-36255-z

**Published:** 2023-01-31

**Authors:** Norman L. Lehman, Nathalie Spassky, Müge Sak, Amy Webb, Cory T. Zumbar, Aisulu Usubalieva, Khaled J. Alkhateeb, Joseph P. McElroy, Kirsteen H. Maclean, Paolo Fadda, Tom Liu, Vineela Gangalapudi, Jamie Carver, Zied Abdullaev, Cynthia Timmers, John R. Parker, Christopher R. Pierson, Bret C. Mobley, Murat Gokden, Eyas M. Hattab, Timothy Parrett, Ralph X. Cooke, Trang D. Lehman, Stefan Costinean, Anil Parwani, Brian J. Williams, Randy L. Jensen, Kenneth Aldape, Akshitkumar M. Mistry

**Affiliations:** 1 Department of Pathology and Laboratory Medicine, University of Louisville, Louisville, KY 40202 USA; 2 Department of Biochemistry and Molecular Genetics, University of Louisville, Louisville, KY 40202 USA; 3 The Brown Cancer Center, University of Louisville, Louisville, KY 40202 USA; 4 Institut de Biologie de l’ENS (IBENS), Inserm, CNRS, École Normale Supérieure, PSL Research University, Paris, France; 5 Department of Biomedical Informatics, The Ohio State University, Columbus, OH 43210 USA; 6 Department of Biomedical Informatics, University of Arkansas for Medical Sciences, Little Rock, AR 72205 USA; 7 Center for Biostatistics, Department of Biomedical Informatics, The Ohio State University, Columbus, OH 43210 USA; 8 NanoString Technologies, Seattle, WA 98109 USA; 9 Department of Cancer Biology, The Ohio State University, Columbus, OH 43210 USA; 10 Solid Tumor Translational Science, The Ohio State University, Columbus, OH 43210 USA; 11 Laboratory of Pathology, Center for Cancer Research, National Cancer Institute, Bethesda, MD 20892 USA; 12 Department of Pathology and Laboratory Medicine, Nationwide Children’s Hospital, Columbus, OH 43205 USA; 13 Department of Pathology, The Ohio State University, Columbus, OH 43210 USA; 14 Department of Pathology, Microbiology and Immunology, Vanderbilt University, Nashville, TN 37232 USA; 15 Department of Pathology and Laboratory Services, University of Arkansas for Medical Sciences, Little Rock, AR 72205 USA; 16 Department of Pathology and Anatomic Sciences, University of Missouri, Columbia, MO 65212 USA; 17 Department of Family and Community Medicine, Contra Costa County Health System, Martinez, CA 94553 USA; 18 Department of Pathology, Banner Gateway Medical Center, MD Anderson Cancer Center, Tempe, AZ 85284 USA; 19 Department of Neurosurgery, University of Louisville, Louisville, KY 40202 USA; 20 Department of Neurosurgery, University of Utah, Salt Lake City, UT 84132 USA; 21 Department of Neurological Surgery, Vanderbilt University, Nashville, TN 37232 USA

**Keywords:** CNS cancer, Neural progenitors, Epigenetics and plasticity, Cancer epigenetics

Correction to: *Nature Communications* 10.1038/s41467-022-29302-8, published online 19 April 2022

The original version of this Article contained an error in Figure 5, in which the indicated developmental timeline origin of outer radial glia was incorrect and the time origin of truncated radial was omitted. This has been corrected in both the PDF and HTML versions of the Article.

