# Supplementary material for: Astroblastomas exhibit radial glia stem cell lineages and differential expression of imprinted and X-inactivation escape genes
Source: Nat Commun. 2022 Apr 19;13:2083. doi: 10.1038/s41467-022-29302-8 (PMC9018799; doi:10.1038/s41467-022-29302-8)
Supplement: Supplementary file 1 — Supplementary Information [file 41467_2022_29302_MOESM1_ESM.pdf]

Supplementary Information File

Supplementary Figure 1. Heatmap with complete gene list for Fig. 1 (RNAseq).

Genes were selected from the most variably expressed and frequently mutated genes. The color scale bar indicates scaled gene expression.

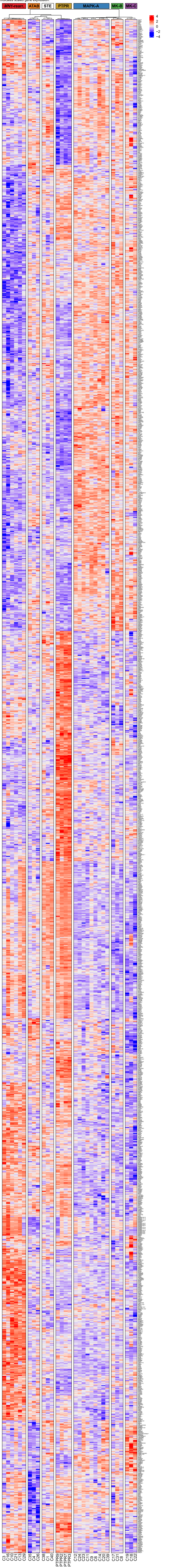

Supplementary Figure 2. Select genes (RNAseq).

Genes were selected from the most variably expressed and frequently mutated genes. The color key and histogram indicates scaled gene expression and correlation distance.

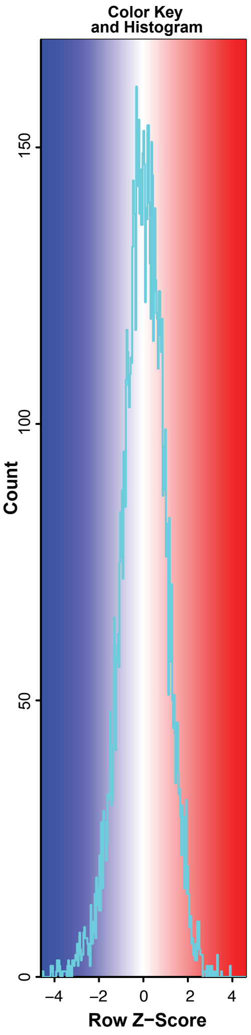

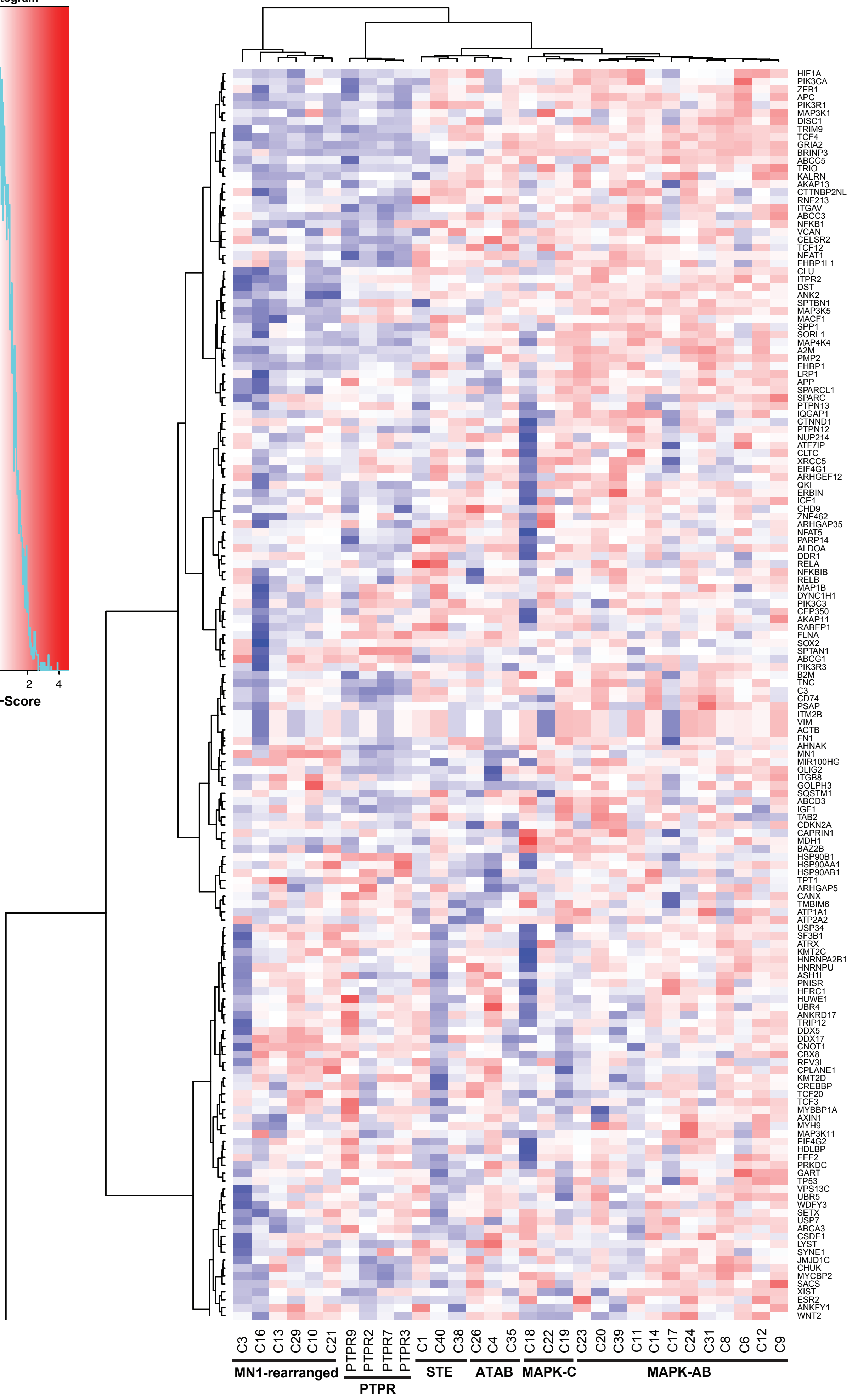

Fig. S2: Select genes (RNAseq) cont.

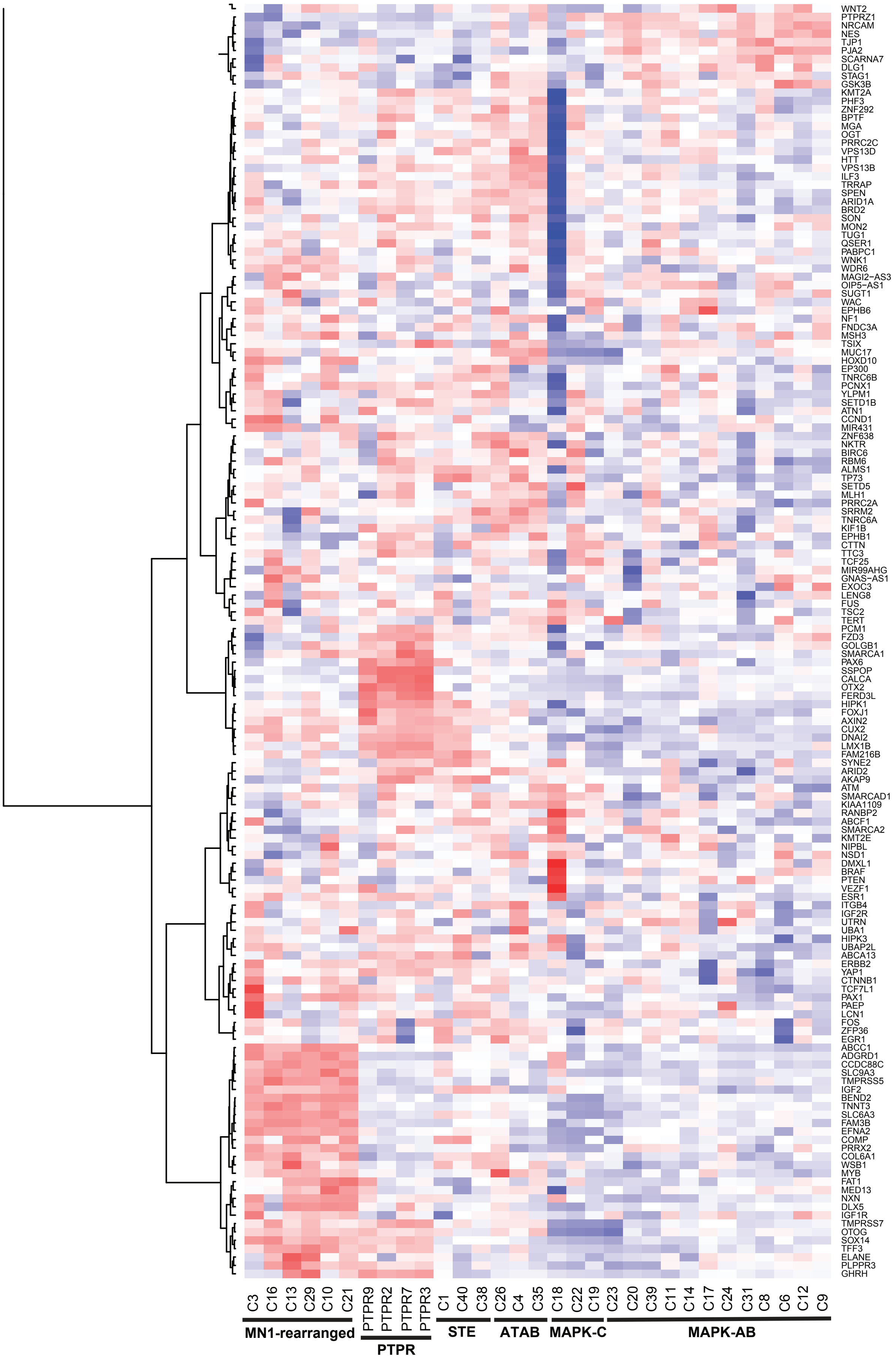

Supplementary Figure 3. Select genes (Affymetrix).

Genes were selected from the most variably expressed and frequently mutated genes.
The color key and histogram indicates scaled gene expression and correlation distance.

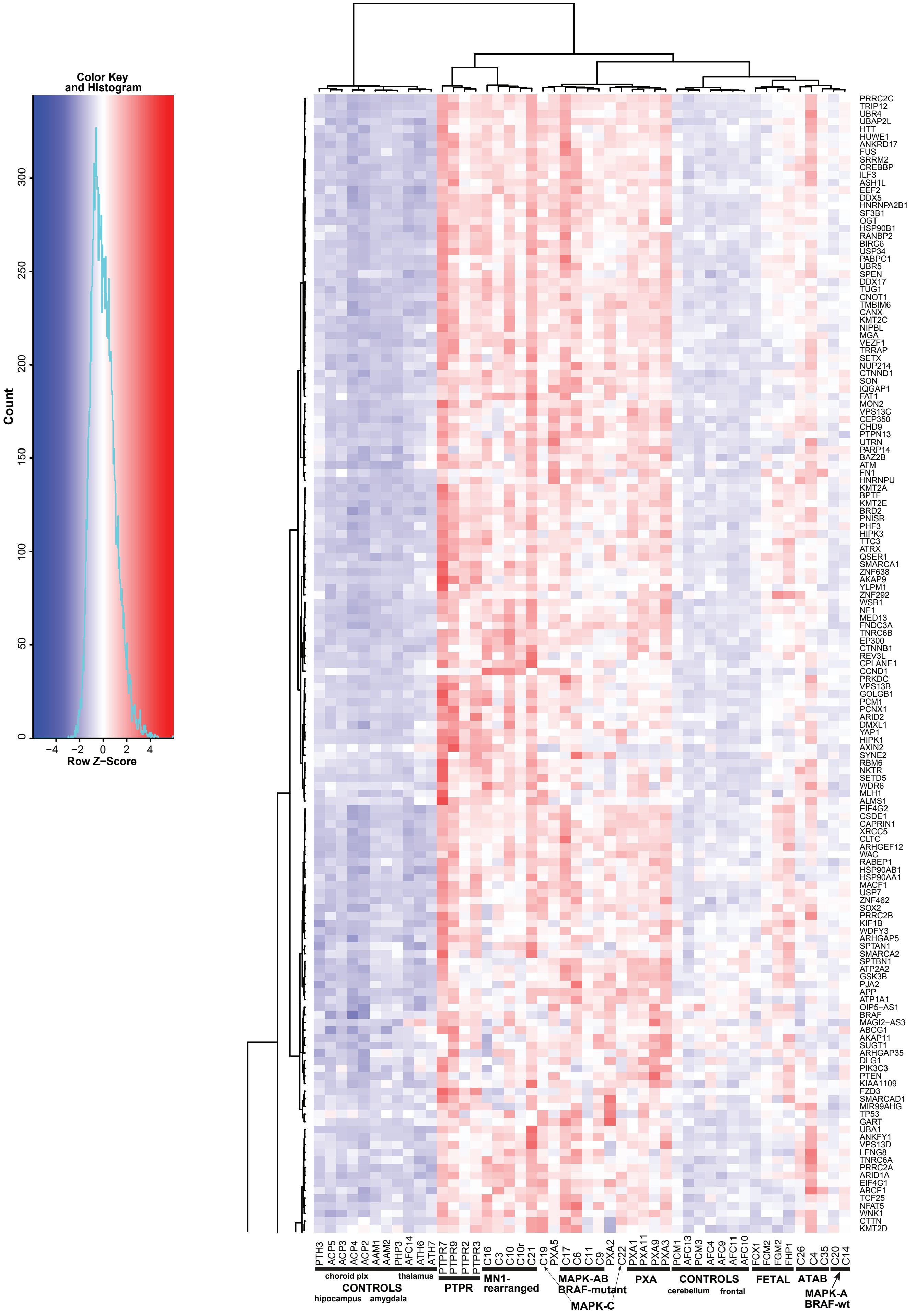

Supplementary Figure 3. Select genes (Affymetrix) cont.

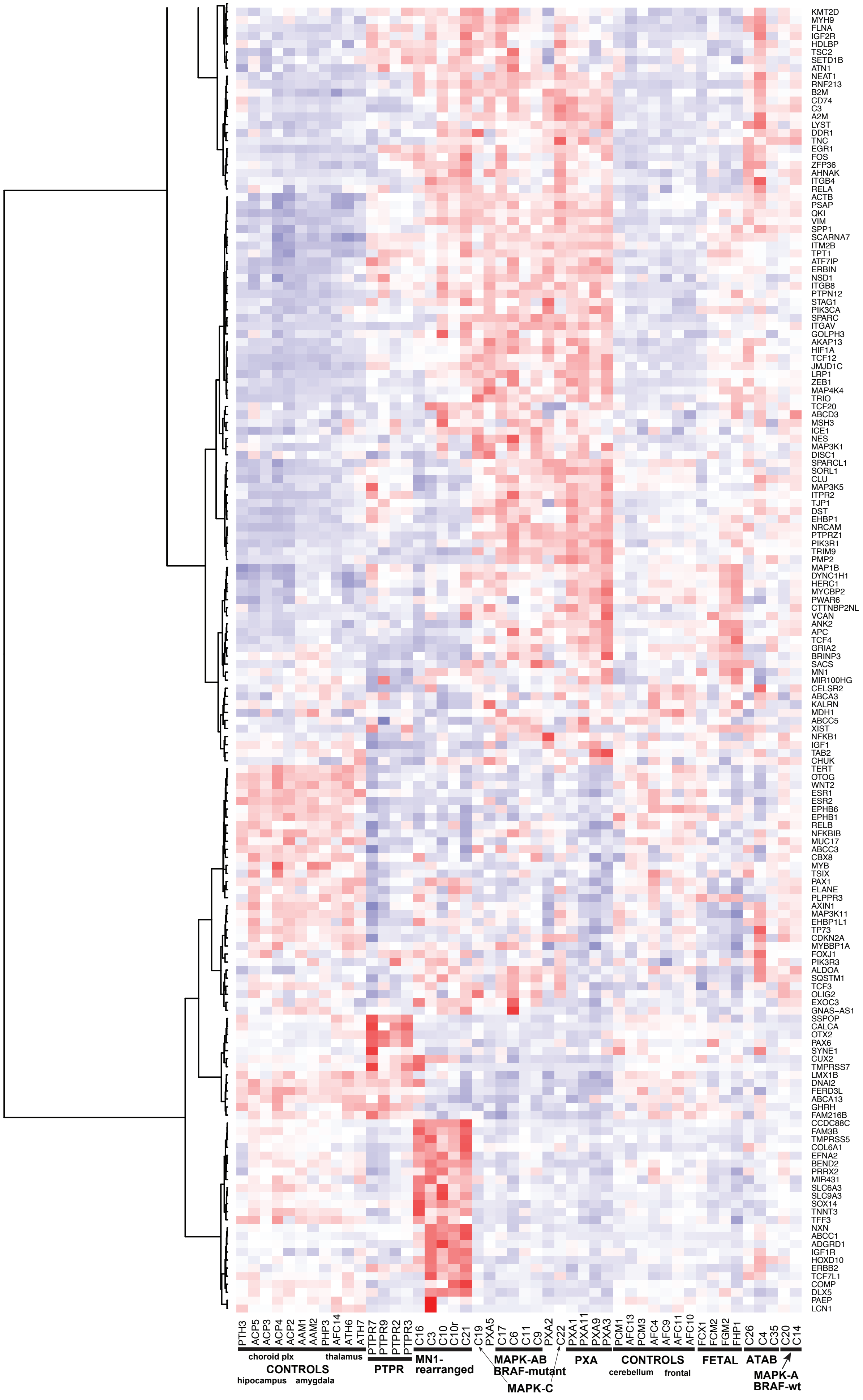

Supplementary Figure 4. Western blots of AB-like tumors validate mRNA data.

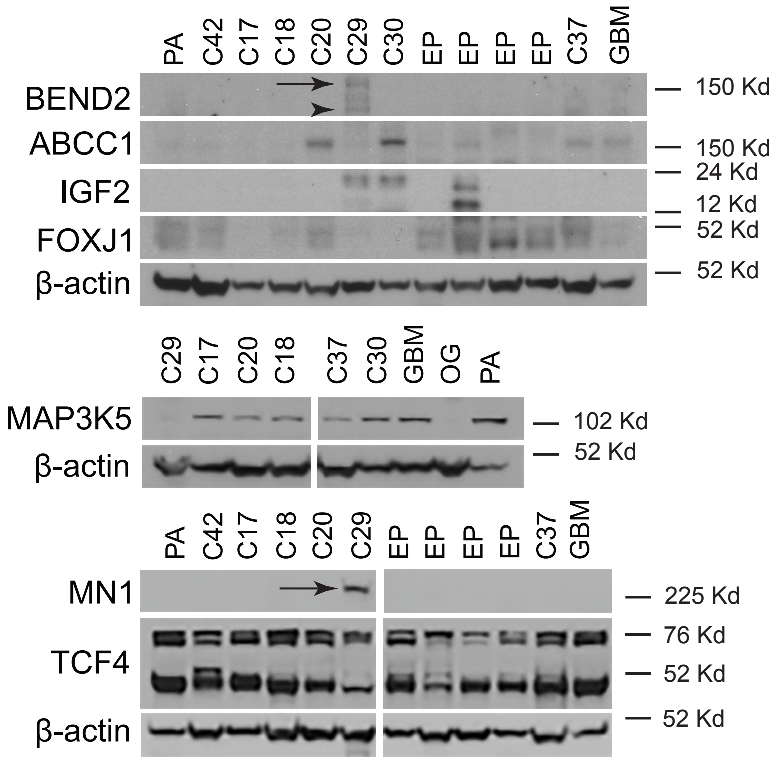

Western blots were performed using tumor lysates from cases with available frozen tumor material. BEND2 protein expression is greatest in the only *MN1-BEND2* tumor with frozen sample available for western blotting, C29 (MW 90KD, arrowhead). A band corresponding to the MN1-BEND2 fusion protein is also only detected in this sample (predicted MW 180 KD, arrow). ABCC1 expression is greatest in C20 and the RELA ependymoma C30. IGF2 protein is seen in the *MN1-BEND2* tumor C29, the RELA ependymoma C30 and a control posterior fossa ependymoma (EP) consistent with expression in tumors of ependymal lineage. FOXJ1 expression is greatest in posterior fossa ependymomas (EP) and non-*MN1-BEND2*, non-*BRAFV600E* tumors C42, C18 and C20, but is also detectable in the *MN1-BEND2* tumor C29. MAP3K5 protein expression is lower in the *MN1-BEND2* tumor C29 compared to MAPK-ABC tumors C17, C18 and C20. MN1 is only detected in the *MN1-BEND2* tumor C29 as the MN1-BEND2 fusion protein (arrow). TCF4 protein expression is strongest in MAPK-ABC tumors. C37 is an otherwise uncharacterized AB-like tumor based on histology alone. Most blots were performed twice with similar results. Additional abbreviations: GBM, glioblastoma; Kd, kilodalton; OG, oligodendroglioma; PA, pilocytic astrocytoma. Original uncropped images of the blots are available in the Source Data File.

Supplementary Figure 5. GSEA heatmaps comparing *MN1-BEND2* to MAPK-ABC tumors for expression of neural stem/progenitor cell marker gens. a NEC, **b** vRG, **c** tRG and **d** oRG genes (RNAseq).

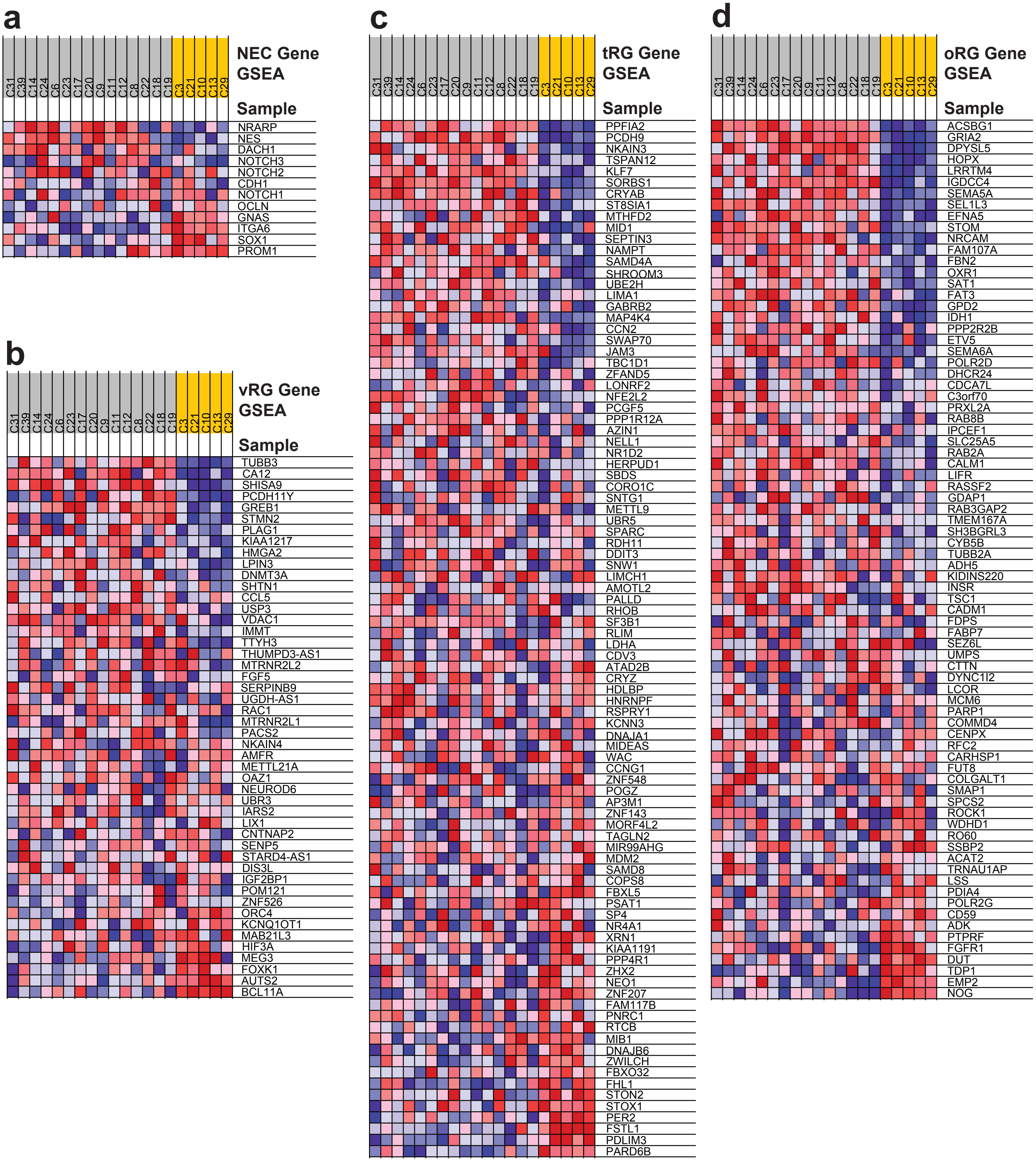

Supplementary Figure 6. GSEA enrichment plots for neural stem/progenitor cell signature gene expression in AB-like tumors and normal and fetal brain control tissue.

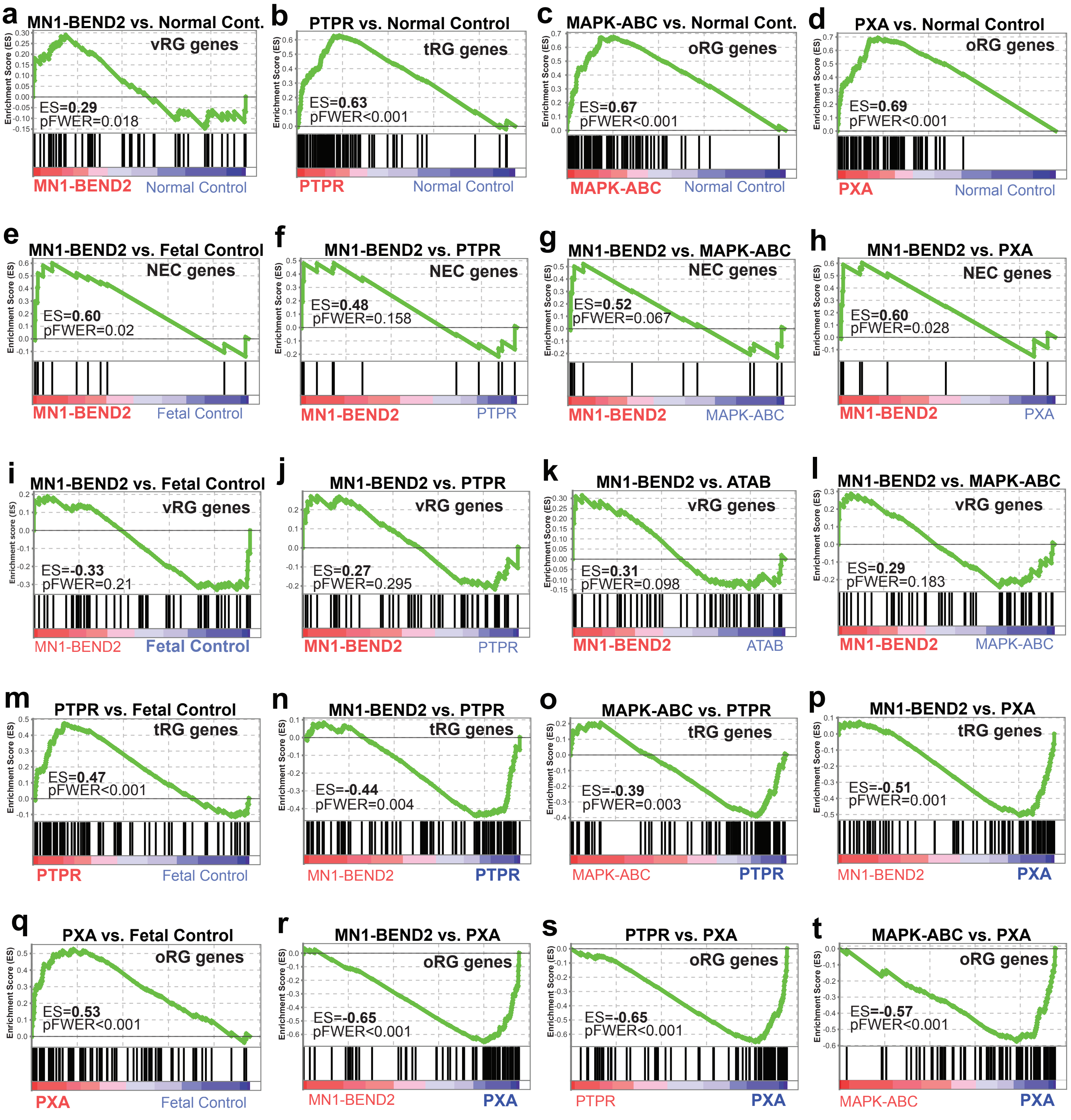

a-d Tumor enrichment for RG signature gene sets compared to normal brain control tissue. **e-h** *MN1-BEND2* tumors are relatively enriched for NEC genes versus control fetal brain tissues and other tumor types. **i-l** *MEN1-BEND2* tumors relatively overexpress vRG genes compared to control fetal brain or other tumor types. **m-p** PTPR show significantly enriched tRG gene expression versus fetal control brain and other tumor types. **q-t** PXA more highly expresses oRG genes compared to fetal control brain tissue and tumor types. The *MN1*-rearranged tumor C16 was included with *MN1-BEND2* tumors. Analyses are two-sided with adjustment for multiple comparisons. Tumors or control tissues comparatively enriched for neural stem cell gene sets are depicted in larger and bold font. Colors are arbitrary. Patient sample numbers: normal control = 18, fetal control = 4, ATAB = 3, MAPK-ABC = 8, *MN1-BEND2* = 4, PTPR = 4, PXA = 6. Source data is available in Supplementary Table 5.

Supplementary Figure 7. Alterations in neural stem cell marker genes. Column colors indicate tumor type groups. Missense = MS, splice region variant = SV, stop gained = SG, stop lost = SL, frameshift = FS, deletion = DEL, insertion = INS, fusion = FU, PT = PTPR. Brown cells indicate fusions detected by only one read. Pink indicates a FathmmMLK score < 0.5, but predicted to be damaging by PolyPhen or deleterious by SIFT. N = 31 patient samples.

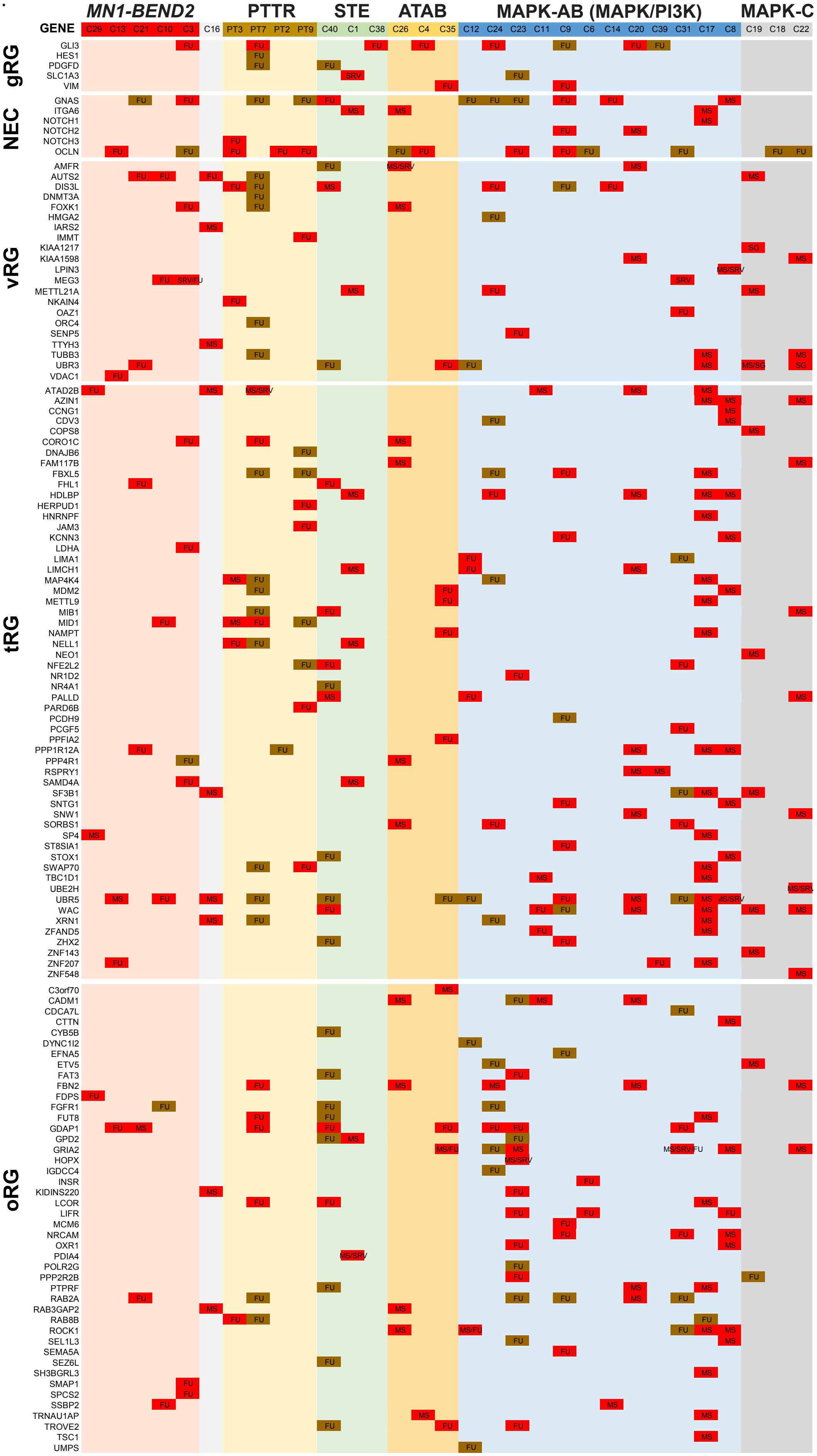

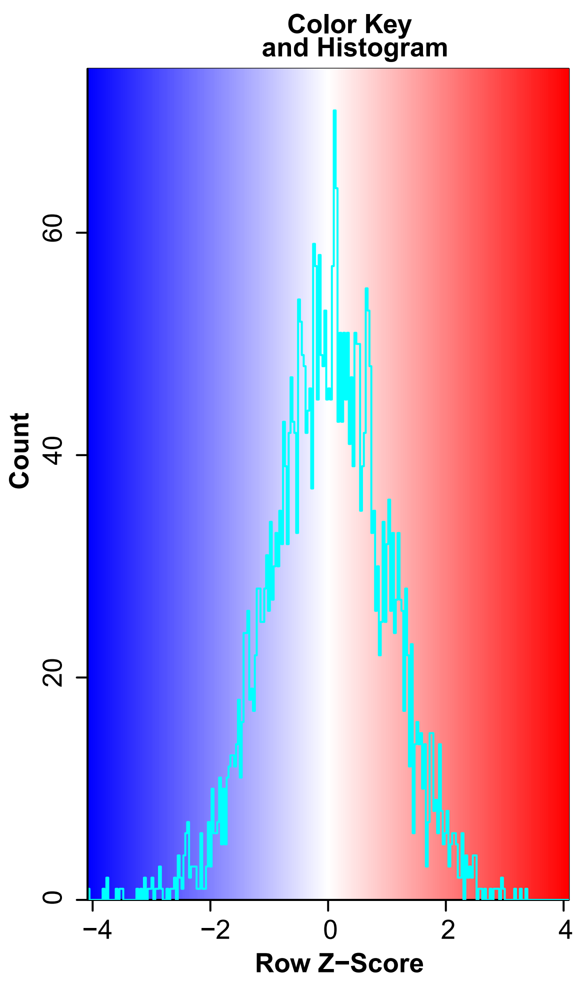

Supplementary Figure 8. Imprinted genes (RNAseq).

The color key and histogram indicates scaled gene expression and correlation distance.

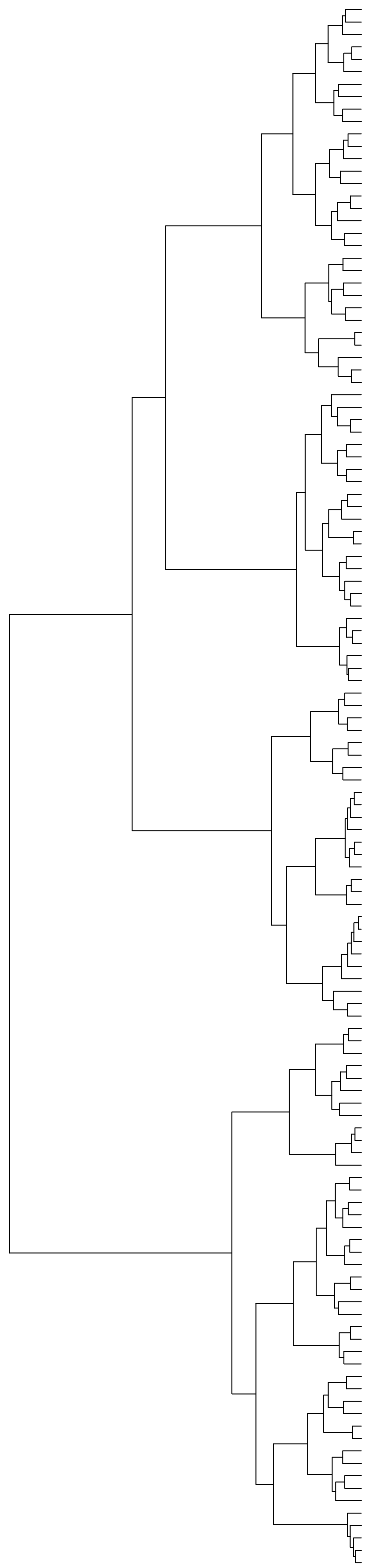

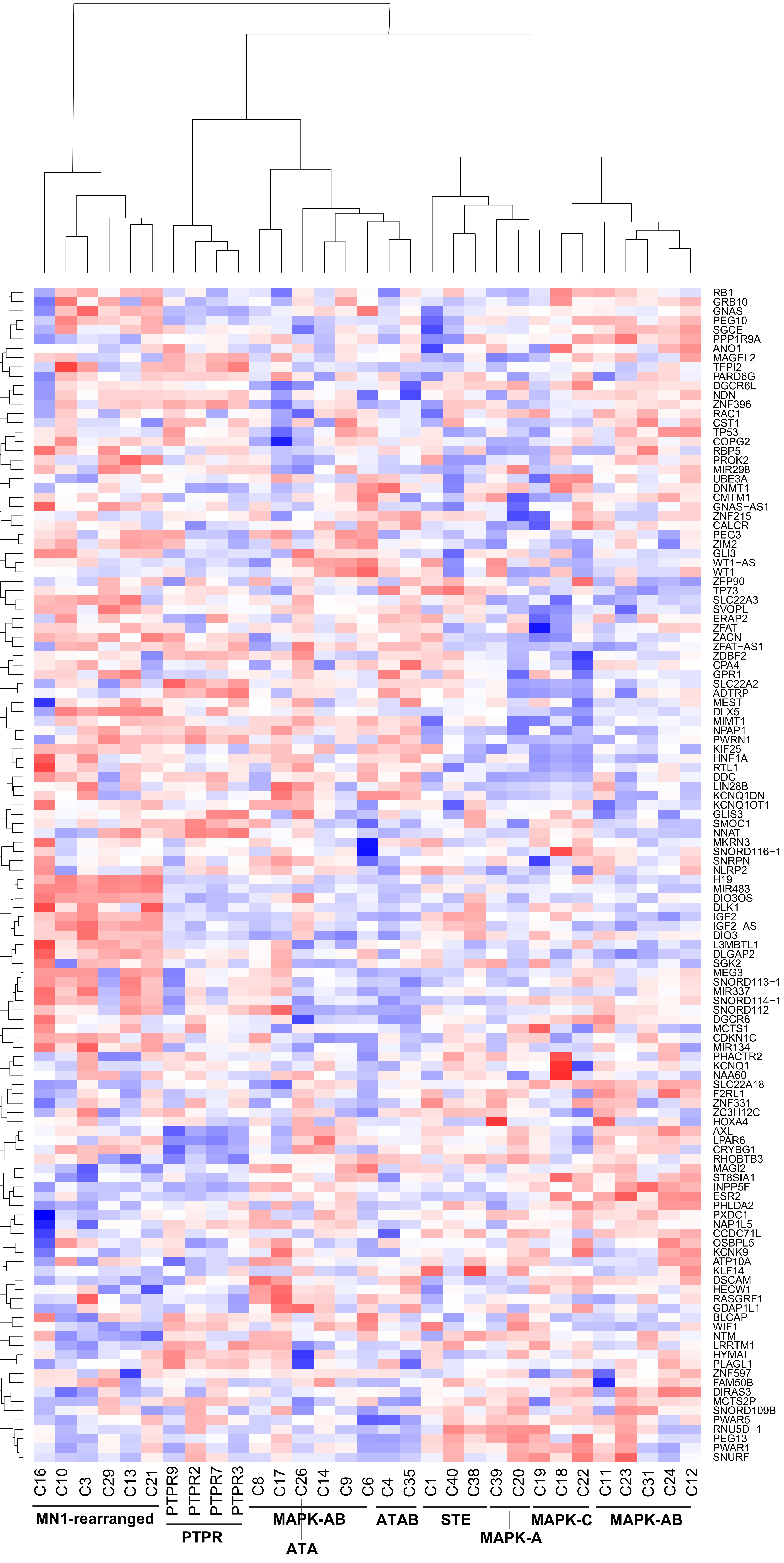

Supplementary Figure 9. Imprinted genes (Affymetrix).

The color key and histogram indicates scaled gene expression and correlation distance.

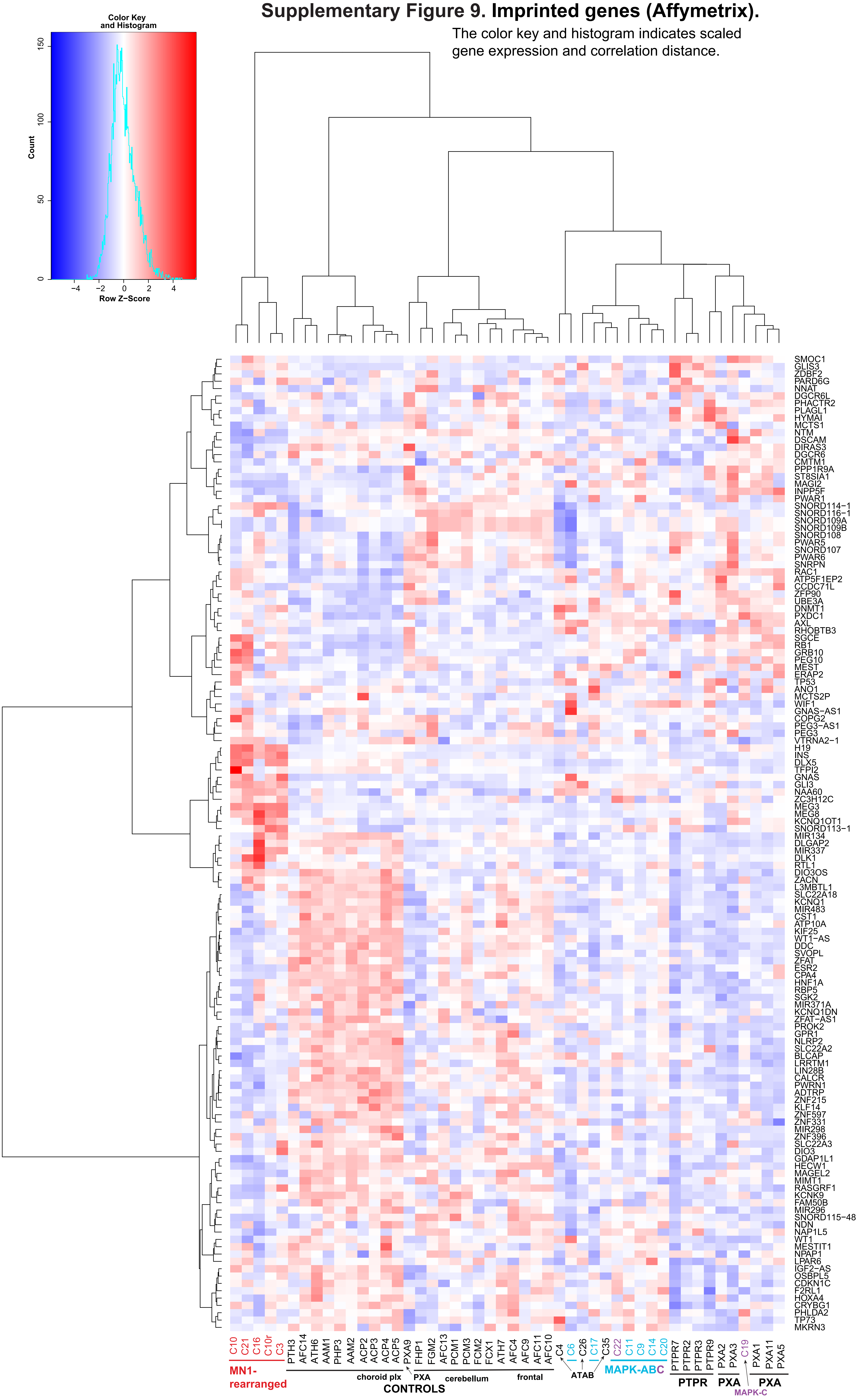

Supplementary Figure 10. Supervised hierarchical clustering of the top 12 differentially expressed miRs in *MN1*-rearranged vs. non-*MN1*-rearranged AB-like tumors.

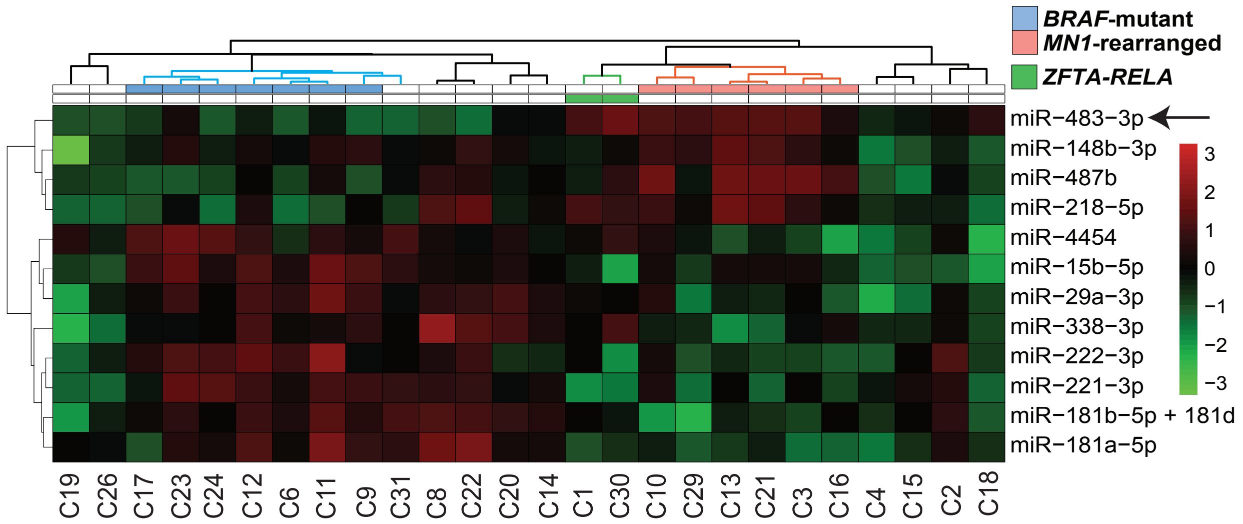

As in RNAseq data, Nanostring miR data revealed that miR 483 (arrow) is comparatively highly expressed in *MN1*- and *RELA*-rearranged tumors. C30 is a *ZFTA-RELA*-harboring STE and C2 is a posterior fossa AB-like tumor, not included in the main analyses due to lack of RNA expression and methylation data, and infratentorial location, respectively. The color scale bar represents scaled miR expression. Source data is available in the Source Data File.

Supplementary Figure 11. Alterations in imprinted genes (a) and XIE and variable XIE genes (b).
Column colors indicate tumor type groups. Missense = MS, splice region variant = SV, stop gained = SG, stop lost = SL, frameshift = FS, deletion = DEL, insertion = INS, fusion = FU, PT = PTPR. Brown cells indicate fusions detected by only one read. Pink indicates a FathmmMLK score < 0.5, but predicted to be damaging by PolyPhen or deleterious by SIFT. N = 31 patient samples.

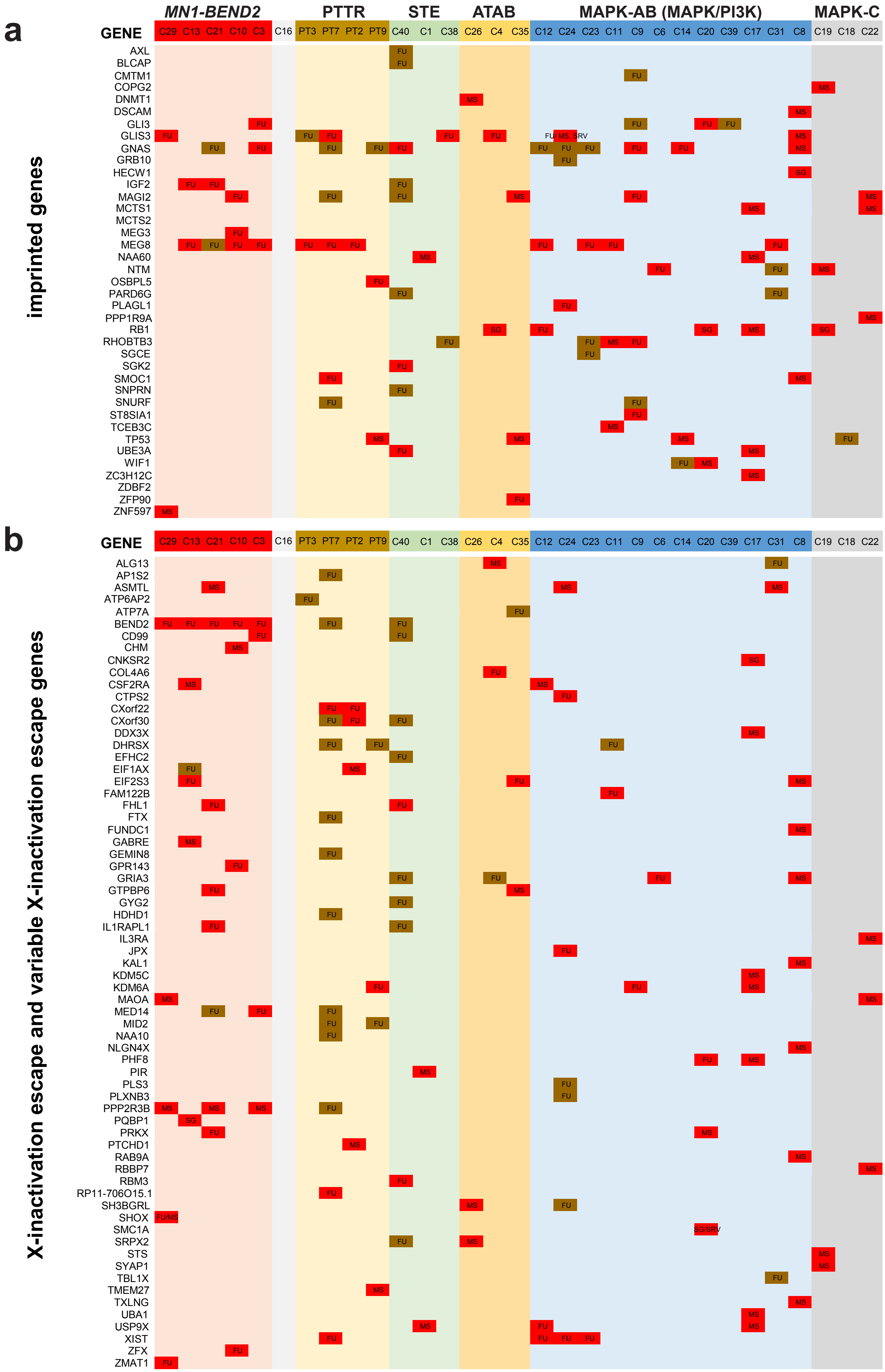

Supplementary Figure 12. XIE and variable XIE Genes (RNAseq).

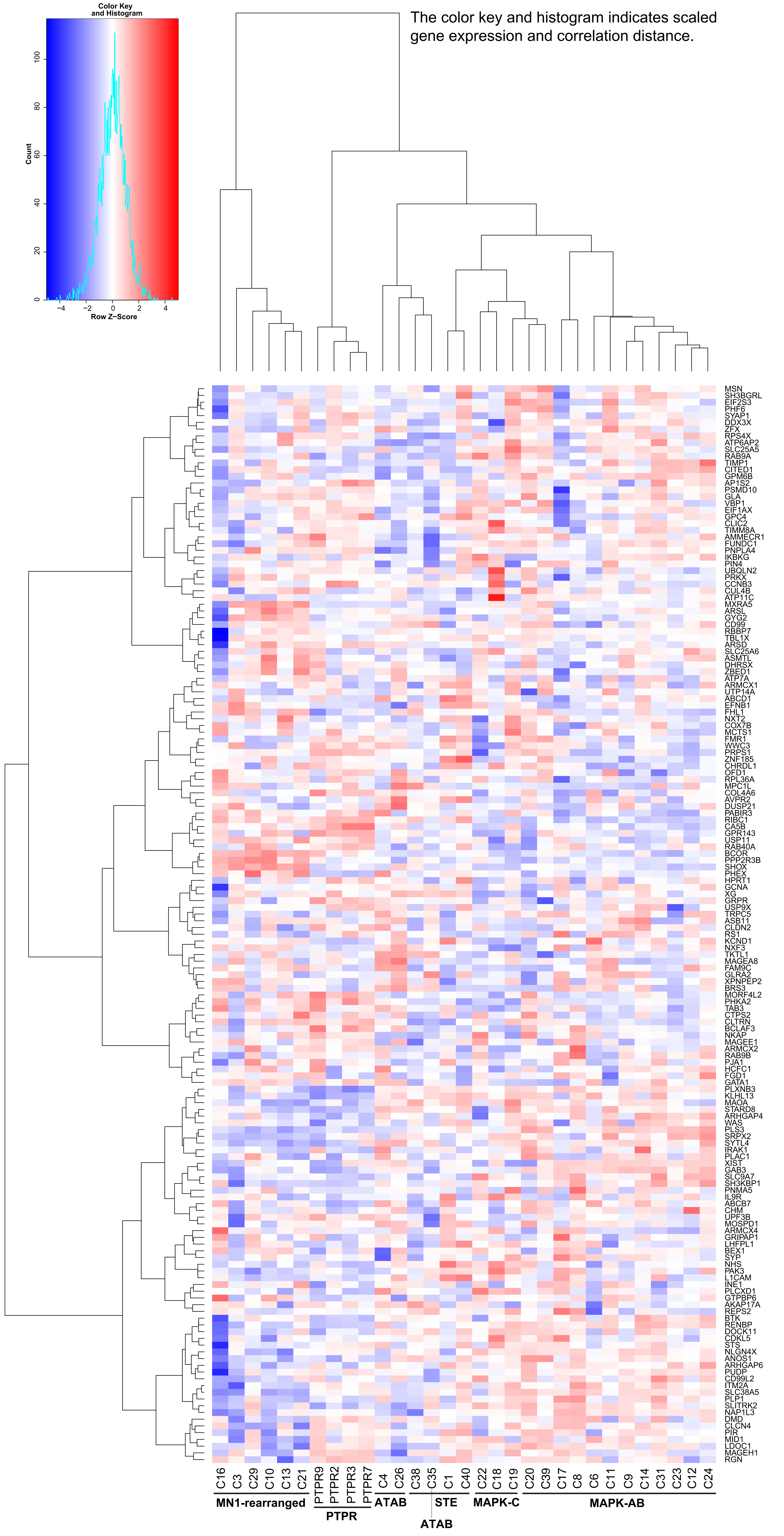

Supplementary Figure 13. XIE and variable XIE Genes (Affymetrix).

The color key and histogram indicates scaled gene expression and correlation distance.

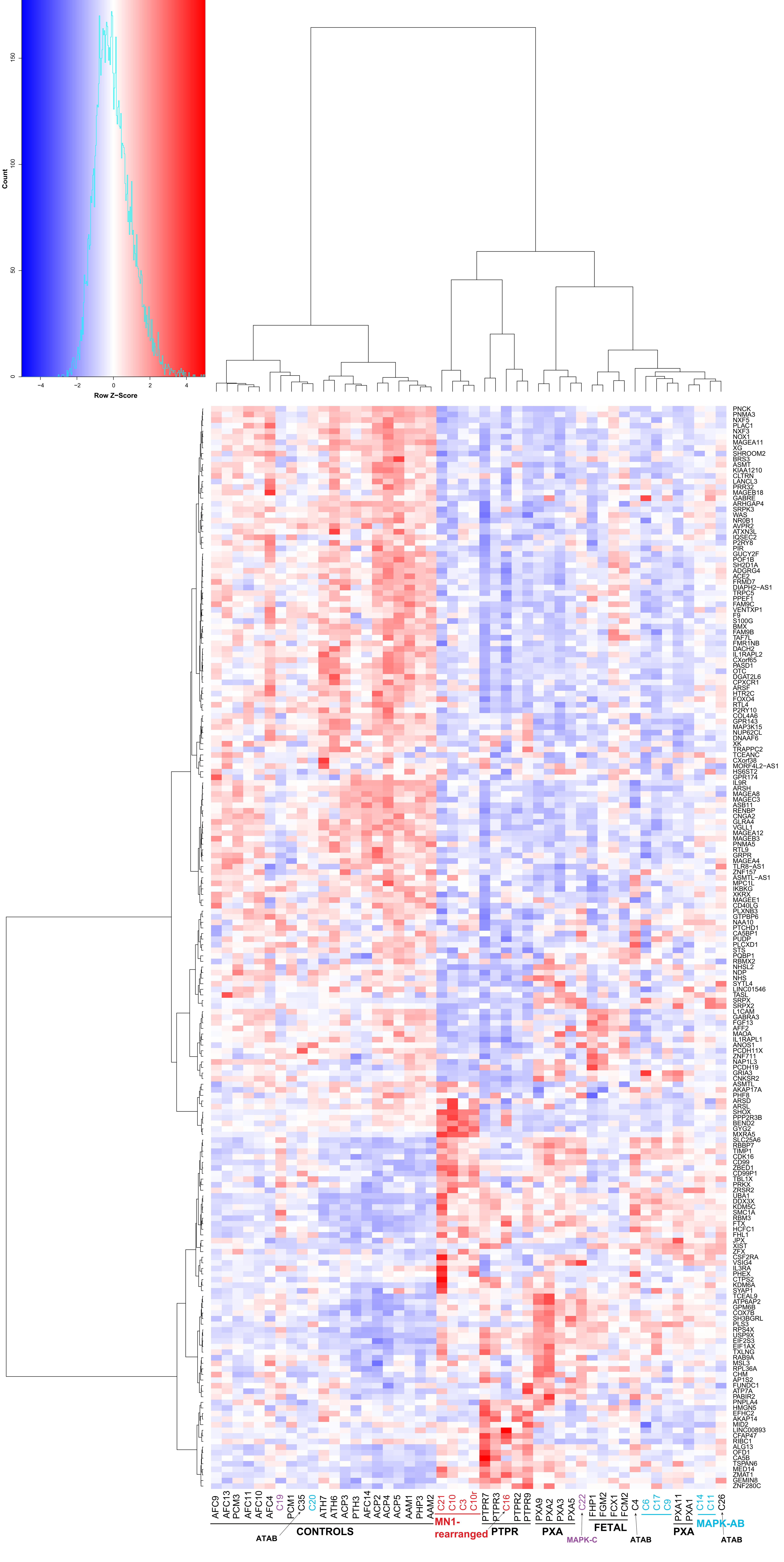

Supplementary Figure 14. Gene expression correlates with gene promoter methylation status.

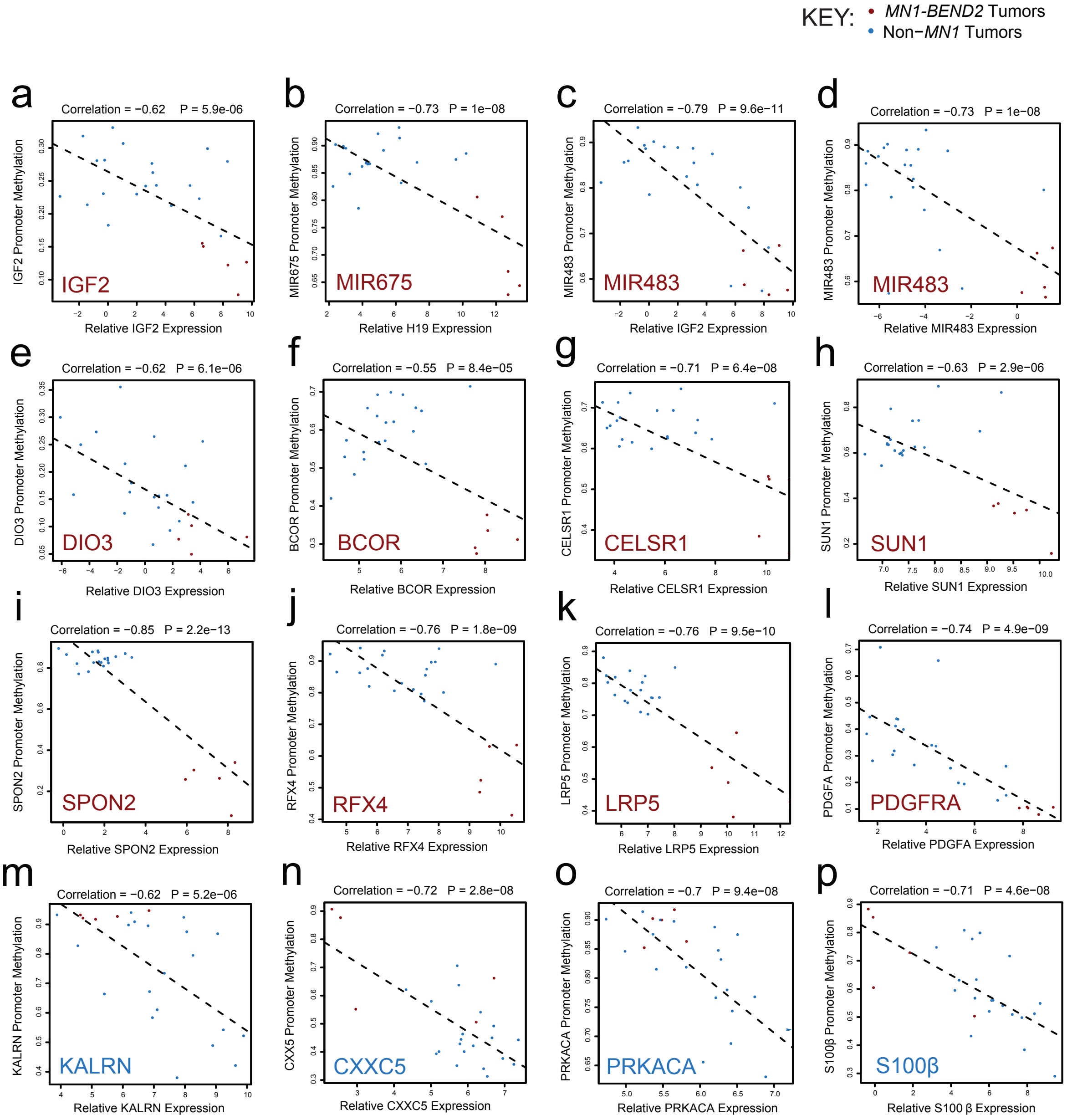

a-e) Examples of imprinted gene expression significantly correlating with promoter methylation status in *MN1-BEND2* tumors. **f)** Expression of *BCOR* correlates with hypomethylation of its promoter in *MN1-BEND2* tumors. **g-h**) Examples of early brain development imprinted early gene expression correlating with promoter methylation in *MN1-BEND2* tumors. **i-l**) Additional developmental genes highly expressed in *MN1-BEND2* tumors. **m**) *KALRN* gene expression correlates with its promoter methylation and is more highly expressed in non-*MN1-BEND2* tumors. **n-p**) Astrocyte-associated gene expression correlates with promoter methylation in non-*MN1-BEND*2 tumors. Analyses are based on RNAseq expres-sion data and Illumina 850K microarray DNA methylation data, and are two-sided without adjustment for multiple comparisons. N = 25 patient samples and P < 0.000001 for all examples shown.
