# Supplementary material for: Astroblastomas exhibit radial glia stem cell lineages and differential expression of imprinted and X-inactivation escape genes
Source: Nat Commun. 2022 Apr 19;13:2083. doi: 10.1038/s41467-022-29302-8 (PMC9018799; doi:10.1038/s41467-022-29302-8)
Supplement: Supplementary file 2 — Description of Additional Supplementary Files [file 41467_2022_29302_MOESM2_ESM.pdf]

File Name: Supplementary Data 1
Description: Gene mutations referenced in manuscript.

File Name: Supplementary Data 2
Description: Gene fusions referenced in manuscript.

File Name: Supplementary Data 3
Description: Signaling and functional pathways of select mutated genes.

File Name: Supplementary Data 4
Description: Gene sets enriched in neural stem cell types.

File Name: Supplementary Data 5
Description: Neural stem cell type GSEA data (Affymetrics).

File Name: Supplementary Data 6
Description: Imprinted and X-inactivation escape (XIE) genes.

File Name: Supplementary Data 7
Description: Imprinted and X- inactivation escape GSEA data (Affymetrics).

File Name: Supplementary Data 8
Description: Summary of MN1-altered and BEND2 fusion gliomas in literature.
