# Supplementary material for: Astroblastomas exhibit radial glia stem cell lineages and differential expression of imprinted and X-inactivation escape genes
Source: Nat Commun. 2022 Apr 19;13:2083. doi: 10.1038/s41467-022-29302-8 (PMC9018799; doi:10.1038/s41467-022-29302-8)
Supplement: Supplementary file 11 — Reporting Summary [file 41467_2022_29302_MOESM11_ESM.pdf]

nature research

Corresponding author(s): Norman L. Lehman

Last updated by author(s): Feb. 12, 2022

Reporting Summary

Nature Research wishes to improve the reproducibility of the work that we publish. This form provides structure for consistency and transparency in reporting. For further information on Nature Research policies, see our Editorial Policies and the Editorial Policy Checklist.

Statistics

For all statistical analyses, confirm that the following items are present in the figure legend, table legend, main text, or Methods section.

n/a	Confirmed	
☒	☐	The exact sample size (*n*) for each experimental group/condition, given as a discrete number and unit of measurement
☒	☐	A statement on whether measurements were taken from distinct samples or whether the same sample was measured repeatedly
☒	☐	The statistical test(s) used AND whether they are one- or two-sided<br>*Only common tests should be described solely by name; describe more complex techniques in the Methods section.*
☒	☐	A description of all covariates tested
☒	☐	A description of any assumptions or corrections, such as tests of normality and adjustment for multiple comparisons
☒	☐	A full description of the statistical parameters including central tendency (e.g. means) or other basic estimates (e.g. regression coefficient) AND variation (e.g. standard deviation) or associated estimates of uncertainty (e.g. confidence intervals)
☒	☐	For null hypothesis testing, the test statistic (e.g. *F*, *t*, *r*) with confidence intervals, effect sizes, degrees of freedom and *P* value noted<br>*Give P values as exact values whenever suitable.*
☐	☒	For Bayesian analysis, information on the choice of priors and Markov chain Monte Carlo settings
☒	☐	For hierarchical and complex designs, identification of the appropriate level for tests and full reporting of outcomes
☒	☐	Estimates of effect sizes (e.g. Cohen's *d*, Pearson's *r*), indicating how they were calculated

Our web collection on statistics for biologists contains articles on many of the points above.

Software and code

Policy information about availability of computer code

Data collection	Primary data generated from primary sample analysis were analyzed.
Data analysis	We used R version 4 and existing packages within Bioconductor for genomic data analysis and as specified in the methods section.

For manuscripts utilizing custom algorithms or software that are central to the research but not yet described in published literature, software must be made available to editors and reviewers. We strongly encourage code deposition in a community repository (e.g. GitHub). See the Nature Research guidelines for submitting code & software for further information.

Data

Policy information about availability of data

All manuscripts must include a data availability statement. This statement should provide the following information, where applicable:

- Accession codes, unique identifiers, or web links for publicly available datasets
- A list of figures that have associated raw data
- A description of any restrictions on data availability

RNAseq, Affymetrix, MethylationEpic BeadChip and Nanostring microRNA raw and processed data are deposited at the public repository Gene Expression Omnibus (GEO) under the accession numbers GSE165351, GSE165813, GSE166569 and GSE196697, respectively. Remaining data are available within the Article, Source Data File, Supplementary Tables and Supplementary Information. Figures 1-5 and 7 and Supplementary Figures 1-3, 5-9 and 11-14 have associated raw data.

Field-specific reporting

Life sciences study design

All studies must disclose on these points even when the disclosure is negative.

Sample size
We studied a rare disease (astroblastoma) and all samples available to the investigators were analyzed.

Data exclusions
Samples not histologically compatible with a diagnosis of astroblastoma were excluded.

Replication
Given the rarity and limited sample volume of this rare disease, replication was not possible. RNA expression was studied by 2 independent methods.

Randomization
n/a

Blinding
n/a

Reporting for specific materials, systems and methods

We require information from authors about some types of materials, experimental systems and methods used in many studies. Here, indicate whether each material, system or method listed is relevant to your study. If you are not sure if a list item applies to your research, read the appropriate section before selecting a response.

Materials & experimental systems

n/a	Involved in the study	
	x	Antibodies
x		Eukaryotic cell lines
x		Palaeontology and archaeology
	x	Animals and other organisms
	x	Human research participants
x		Clinical data
x		Dual use research of concern

Methods

n/a	Involved in the study	
x		ChIP-seq
x		Flow cytometry
x		MRI-based neuroimaging

Antibodies

Antibodies used
GFAP clone EP672Y (Cell Marque 258R-16, lot 32653),MN1 (Proteintech 24697-1-AP, lot 00021048) IGF2 (Abcam ab9574, lots GR31975-61 and GR31975-64) Targeted polyglutamylated tubulin (Adipogen AG-20B-0020-C100), BEND2 (Invitrogen PA5-31747, lot UA2709054B), IGF2 (Abcam ab262713, lot GR3295940-1), MAP3K5 (Millipore MABC632, lot VP1812286), ABCC1 (Abcam ab24102, lot GR3247402-6), FOXJ1 (Novus NBP1-87928, lot H119212), MN1 (Invitrogen PA5-38666, lot UD2755192), TCF4 (Proteintech 22337-1-AP, lot 00050018), β-actin (Sigma A2228, lot 085M4754V).

Validation
Antibodies used for immunohistochemistry were validated by titering using human autopsy brain tissue. Antibodies for immunofluorescence were validated by titering using mouse embryo and newborn pup brain sections. Antibodies for western blotting were validated with human glioma tumor lysates.

Animals and other organisms

Policy information about studies involving animals; ARRIVE guidelines recommended for reporting animal research

Laboratory animals
Mice used in this study are Centrin2-GFP (CB6-Tg(CAG-EGFP/CETN2)3- 4Jgg/J; The Jackson Laboratory). Animals were group-housed with free access to food and water in controlled temperature conditions (room temperature controlled at 21–22 °C, humidity between 40 and 50%), and exposed to a conventional 12-h light/dark cycle. Experiments were performed on embryos at embryonic (E) days E16, and neonate pups at postnatal day 0–10 (P0–10) of both sexes. All efforts were made to reduce animal suffering and minimize the number of animals, in compliance with all relevant ethical regulations for animal testing and research. Animal brains were dissected out in cold PBS from embryos or newborn animals euthanized by decapitation.

Wild animals
No wild animals were used in the study.

Field-collected samples
No field-collected samples were used in the study.

Ethics oversight
Animal studies were performed in accordance with the guidelines of the European Community and French Ministry of Agriculture and were approved by the "Direction départementale de la protection des populations - Paris (authorization 93432017022117065610). All procedures were approved by the Ethical Committee CEEA-005 Charles Darwin (authorization 9343-201702211706561) and conducted in accordance with EU Directive 2010/63/EU.

Note that full information on the approval of the study protocol must also be provided in the manuscript.

Human research participants

Policy information about studies involving human research participants

Population characteristics
Tumor samples were collected restrospectively from archived FFPE tissue following IRB approval.

Recruitment
n/a

Ethics oversight
The study was performed with IRB approval.

Note that full information on the approval of the study protocol must also be provided in the manuscript.

Clinical data

Policy information about clinical studies

All manuscripts should comply with the ICMJE guidelines for publication of clinical research and a completed CONSORT checklist must be included with all submissions.

Clinical trial registration	n/a
Study protocol	n/a
Data collection	n/a
Outcomes	n/a
